# A *DNAH17* missense variant causes flagella destabilization and asthenozoospermia

**DOI:** 10.1084/jem.20182365

**Published:** 2019-10-28

**Authors:** Beibei Zhang, Hui Ma, Teka Khan, Ao Ma, Tao Li, Huan Zhang, Jianing Gao, Jianteng Zhou, Yang Li, Changping Yu, Jianqiang Bao, Asim Ali, Ghulam Murtaza, Hao Yin, Qian Gao, Xiaohua Jiang, Feng Zhang, Chunyu Liu, Ihsan Khan, Muhammad Zubair, Hafiz Muhammad Jafar Hussain, Ranjha Khan, Ayesha Yousaf, Limin Yuan, Yan Lu, Xiaoling Xu, Yun Wang, Qizhao Tao, Qiaomei Hao, Hui Fang, Hongtao Cheng, Yuanwei Zhang, Qinghua Shi

**Affiliations:** 1 The First Affiliated Hospital of University of Science and Technology of China, Hefei National Laboratory for Physical Sciences at Microscale, University of Science and Technology of China-Shenyang Jinghua Hospital Joint Center for Human Reproduction and Genetics, Chinese Academy of Sciences (CAS) Key Laboratory of Innate Immunity and Chronic Diseases, School of Life Sciences, CAS Center for Excellence in Molecular Cell Science, Collaborative Innovation Center of Genetics and Development, University of Science and Technology of China, Hefei, China; 2 Obstetrics and Gynecology Hospital, State Key Laboratory of Genetic Engineering at School of Life Sciences, Institute of Reproduction and Development, Fudan University, Shanghai, China; 3 Key Laboratory of Reproduction Regulation of National Population and Family Planning Commission, Collaborative Innovation Center of Genetics and Development, Fudan University, Shanghai, China; 4 Shanghai Key Laboratory of Female Reproductive Endocrine Related Diseases, Shanghai, China; 5 Analysis and test center, Co-Innovation Center for Modern Production Technology of Grain Crops, Yangzhou University, Yangzhou, China; 6 Department of Respiration, The First Affiliated Hospital of University of Science and Technology of China, Division of Life Sciences and Medicine, University of Science and Technology of China, Hefei, China

## Abstract

Asthenozoospermia is a common cause of male infertility, but its etiology remains incompletely understood. We recruited three Pakistani infertile brothers, born to first-cousin parents, displaying idiopathic asthenozoospermia but no ciliary-related symptoms. Whole-exome sequencing identified a missense variant (c.G5408A, p.C1803Y) in *DNAH17*, a functionally uncharacterized gene, recessively cosegregating with asthenozoospermia in the family. *DNAH17*, specifically expressed in testes, was localized to sperm flagella, and the mutation did not alter its localization. However, spermatozoa of all three patients showed higher frequencies of microtubule doublet(s) 4–7 missing at principal piece and end piece than in controls. Mice carrying a homozygous mutation (*Dnah17^M/M^*) equivalent to that in patients recapitulated the defects in patients’ sperm tails. Further examinations revealed that the doublets 4–7 were destabilized largely due to the storage of sperm in epididymis. Altogether, we first report that a homozygous *DNAH17* missense variant specifically induces doublets 4–7 destabilization and consequently causes asthenozoospermia, providing a novel marker for genetic counseling and diagnosis of male infertility.

## Abstract (precis)

Using mice modelling patients’ variant, this study demonstrates that a homozygous *DNAH17* missense variant causes asthenozoospermia and specifically destabilizes microtubule doublets 4–7 in flagella, which could be largely due to the storage of sperm in epididymis.

## Introduction

According to the World Health Organization (WHO), men whose ejaculates have <32% progressively motile sperm are diagnosed with asthenozoospermia (WHO, 2010). Asthenozoospermia is one of the major causes of male infertility. Isolated asthenozoospermia accounts for 19% of all infertile men, and oligo- and/or terato-asthenozoospermia could account for 63% of all infertile men (Curi et al., 2003). Numerous factors, like lifestyle, pollutants, prolonged sexual abstinence, partial blockage of seminal tract, varicocele, and infection, have been reported as causes of asthenozoospermia (Adams et al., 2014; Ortega et al., 2011; Salas-Huetos et al., 2017). Nonetheless, the genetic factors underlying asthenozoospermia remain largely unknown.

Axoneme is the core structure of sperm flagellum, presenting throughout the flagellar length. The axoneme is typically composed of 9+2 microtubules, where a central pair of microtubules is surrounded by nine peripheral microtubule doublets (MTDs) in the fixed order (Inaba, 2011). Axonemal dyneins are a pair of projecting “hooks,” consisting of an inner and an outer dynein arm (IDA and ODA, respectively), which are attached to each of the nine MTDs (Kikkawa, 2013). IDAs and ODAs are structural subunits of axoneme and essential for generating beating forces of sperm flagella (Gibbons, 1963; Summers and Gibbons, 1971). Each dynein arm is composed of several light chain proteins, at least two intermediate chain proteins, and at least two heavy chain proteins that hydrolyze ATPs for microtubule sliding (Inaba, 2011; Roberts et al., 2013).

Heavy chains, also known as dynein axonemal heavy chains (DNAHs), comprise 13 members (DNAH1–3, 5–12, 14, and 17) in humans (Pazour et al., 2006). Disruptions in DNAHs, such as *DNAH5* (Hornef et al., 2006; Olbrich et al., 2002), *DNAH6* (Li et al., 2016), *DNAH9* (Fassad et al., 2018; Loges et al., 2018), and *DNAH11* (Bartoloni et al., 2002; Knowles et al., 2012; Lucas et al., 2012; Schwabe et al., 2008), are known to cause, or are associated with, primary ciliary dyskinesia (PCD), a genetically heterogeneous disorder that is characterized by chronic airway diseases, left–right laterality disturbances, and male infertility (Leigh et al., 2009). So far, mutations in only *DNAH1* or *DNAH9* have been described in patients with asthenozoospermia. Patients harboring biallelic *DNAH1* mutations were infertile and displayed impaired sperm motility and multiple morphological abnormalities of sperm flagella (MMAF), including absent, bent, short, coiled, and irregular-caliber flagella (Coutton et al., 2018; Ben Khelifa et al., 2014; Sha et al., 2017; Tang et al., 2017; Wang et al., 2017); an infertile patient with two homozygous *DNAH9* mutations displayed markedly reduced sperm counts and motility, as well as absence of morphologically normal sperm (i.e., oligoasthenozoospermia; Fassad et al., 2018), whereas their functional roles in maintaining sperm motility and flagellar structure have not been fully understood. Interestingly, *DNAH17*, encoding an ODA component, showed testis-specific mRNA expression in humans (Milisav and Affara, 1998) but has not yet been functionally characterized.

In this study, we recruited three primary infertile patients from Pakistan, born to a consanguineous union and suffering from asthenozoospermia with no MMAF-like phenotype or ciliary-related symptoms. Through whole-exome sequencing (WES) and Sanger sequencing, we identified a homozygous missense variant in *DNAH17* recessively cosegregating with asthenozoospermia in this family. Further analyses of spermatozoa from patients and functional studies in mice carrying a *Dnah17* mutation equivalent to that in patients collectively demonstrated that the *DNAH17* variant specifically induces doublets 4–7 destabilization during sperm storage in epididymides and thus causes asthenozoospermia, signifying that DNAH17 is the first DNAH protein implicated in stabilizing flagellar structure.

## Results

### Three asthenozoospermic patients born to a consanguineous union

**Figure 1.**
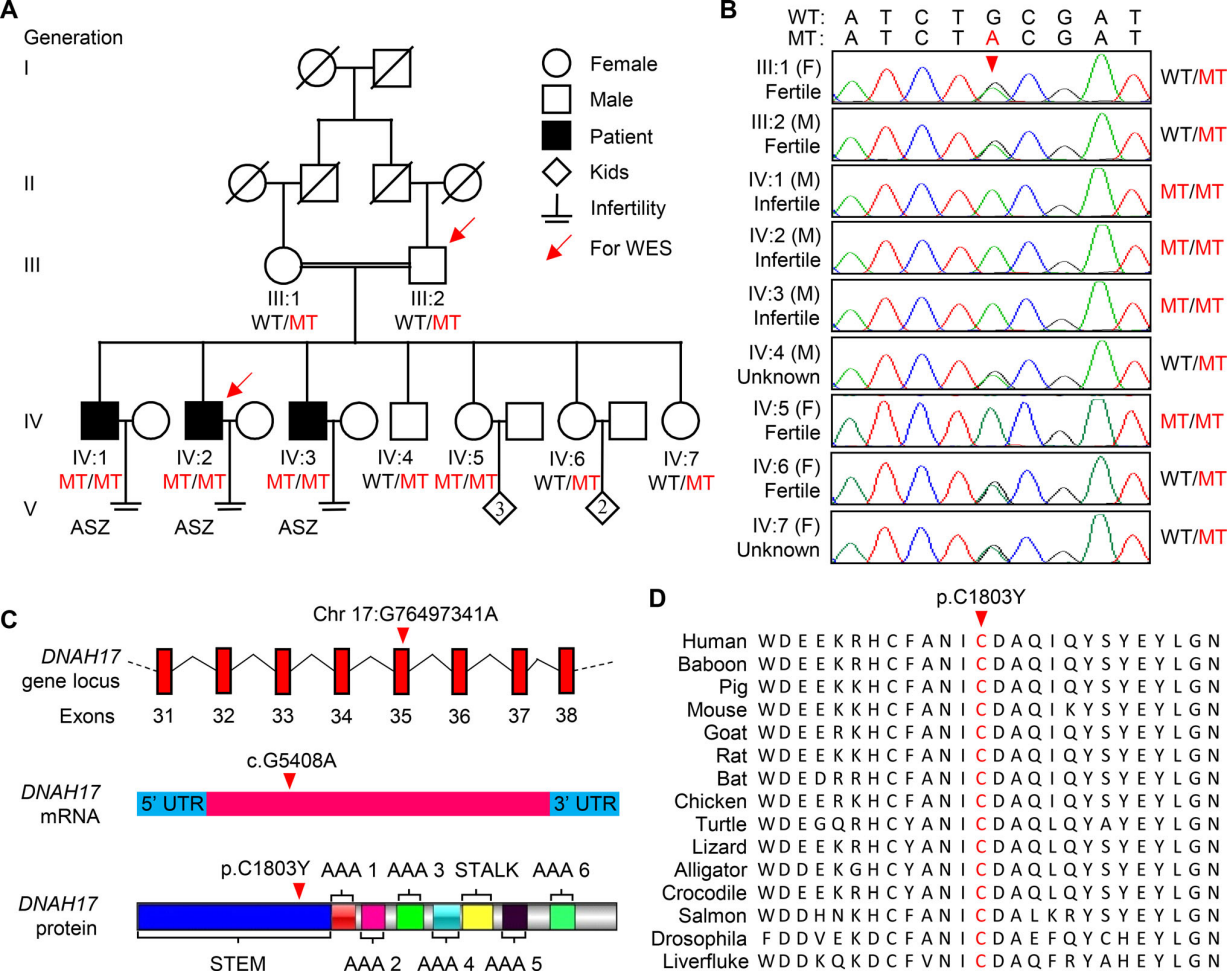
**A *DNAH17* missense variant in a consanguineous Pakistani family with asthenozoospermia. (A)** Pedigree of the consanguineous family with three asthenozoospermia patients (IV:1, IV:2, and IV:3). Arrows point to the two individuals for whom WES was performed. Slashes denote deceased family members, and the double horizontal lines represent consanguineous marriage. ASZ, asthenozoospermia. **(B)** Chromatograms of the *DNAH17* missense mutation (g.G78136A) in genomic DNA from all the available family members. F, female; M, male. **(C)** The *DNAH17* mutation occurs in exon 35 and causes a G-to-A substitution at cDNA (NCBI reference sequence no. NM_173628) nucleotide position 5408, replacing cysteine (C) with tyrosine (Y) at amino acid 1803 in the DNAH17 protein (UniProt accession no. Q9UFH2). **(D)** Sequence alignment shows conservation of the affected amino acid (cysteine) across different organisms. Arrowheads, the mutation site; WT, the wild-type allele; MT, the mutant allele; UTR, untranslated region.

This study was performed on a family with male infertility originating from Pakistan (Fig. 1 A). The parents (III:1 and III:2) were first-degree cousins and gave birth to three daughters and four sons. Two sisters (IV:5, 42 yr old and IV:6, 27 yr old) had three and two children, respectively, and the youngest sister (IV:7, 25 yr old), who had normal menstrual cycles, was unmarried. Among the four brothers, one (IV:4, 28 yr old) was unmarried; the other three, IV:1 (43 yr old), IV:2 (41 yr old), and IV:3 (29 yr old), had been married for 20, 17, and 11 yrs, respectively, but all were infertile. They did not have any history of drinking, smoking, exposure to toxic chemicals, or any symptoms of ciliary-related diseases and were physically normal with respect to height, weight, external genitalia, and testicular size. Semen analyses of patients revealed that semen volumes, sperm concentrations, and percentages of morphologically normal sperm fell within the normal ranges (WHO, 2010). However, all three patients exhibited reduced sperm motility, with ≤25.0% of motile sperm and ≤17.5% progressively motile sperm. Hence, they were diagnosed with asthenozoospermia. Patients’ clinical characteristics are summarized in Table 1.

**Table 1.** Clinical characteristics of patients

	Reference values^a^	IV:1	IV:2	IV:3
Genotype		MT/MT	MT/MT	MT/MT
Age (years)^b^		43	41	29
Years of marriage^c^		20	17	11
Height/weight (cm/kg)		182.9/70.0	167.6/70.0	167.6/50.0
**Semen parameters**				
Semen volume (ml)	>1.5	3.3 ± 0.9	2.0 ± 1.0	3.5 ± 0.7
Semen pH	Alkaline	Alkaline	Alkaline	Alkaline
Sperm concentration (10^6^/ml)	>15	18.0 ± 1.4	30.0 ± 0	17.3 ± 1.6
Morphologically normal sperm (%)	>4	76.8 ± 2.8	83.8 ± 0.3	78.4 ± 0.5
Motile sperm (%)	>40	11.5 ± 4.6	25.0 ± 10.6	15.1 ± 5.0
Progressively motile sperm (%)	>32	5.5 ± 1.8	17.5 ± 8.8	9.4 ± 3.1

Two independent experiments were performed. Data are presented as mean ± SEM. MT, the mutant allele.

a Reference values were published in WHO (2010).

b The current ages.

c The current years of marriage.

### Identification of a candidate pathogenic variant in *DNAH17*

**Figure S1.**
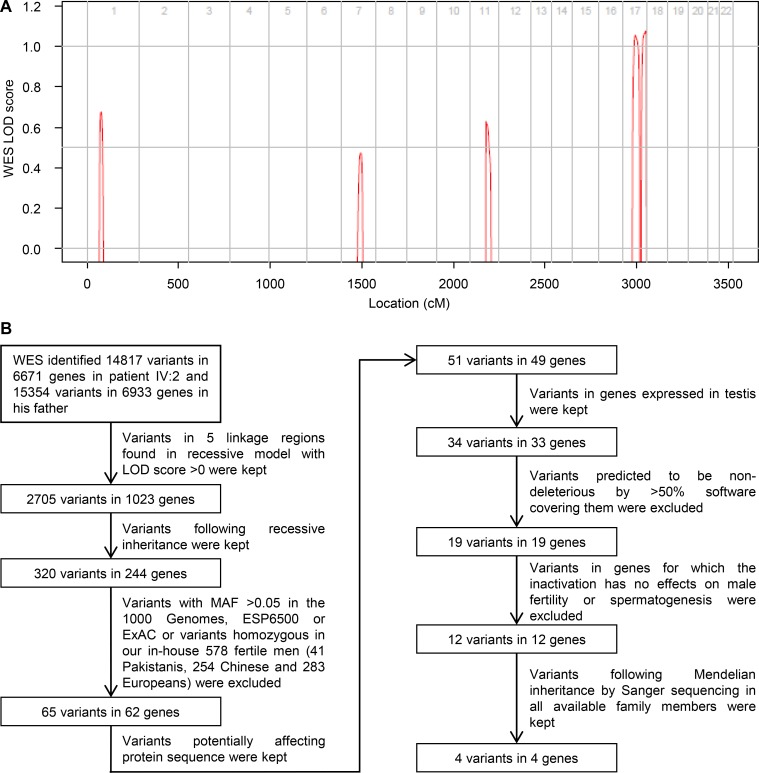
**WES data analysis. (A)** Genome-wide logarithm of the odds scores using WES-derived genotypes for the family. **(B)** WES data analysis pipeline. LOD, logarithm of the odds; MAF, minor allele frequency.

To understand the genetic cause of asthenozoospermia in this family, we performed WES for patient IV:2 and his father. Following a pipeline of WES data analysis (Fig. S1), 12 variants in 12 genes were retained. Subsequent Sanger sequencing on genomic DNA from all the available family members (III:1, III:2, and IV:1–7) verified four variants in four genes (*DNAH17*, *GPS1*,* HID1*, and *USP36*) recessively coinherited with asthenozoospermia (Fig. 1 B and Fig. S1 B). *GPS1*, *HID1*, and *USP36* are annotated ubiquitously expressed in various tissues (Zhang et al., 2013; *DNAH17* mRNA exhibits testis-specific expression [Milisav and Affara, 1998]). Given that our patients did not show any other symptoms except asthenozoospermia, the homozygous missense variant (c.G5408A) in *DNAH17* was favorably presumed to be responsible for the diminished sperm motility in the patients.

The *DNAH17* c.G5408A occurred in exon 35 and caused a G-to-A substitution at cDNA (NCBI reference sequence no. NM_173628) nucleotide position 5408, predicted to replace cysteine (C) by tyrosine (Y) at amino acid position 1803 (p.C1803Y; Fig. 1 C). The altered amino acid is located in the N-terminal stem region of DNAH17, which is known to interact with other dynein components. Phylogenic analysis revealed that the altered amino acid was conserved from lower to higher organisms (Fig. 1 D). All these findings suggest that the homozygous *DNAH17* variant (c.G5408A) could be pathogenic for asthenozoospermia in this family.

### Generation and validation of the anti-DNAH17 antibody

**Figure 2.**
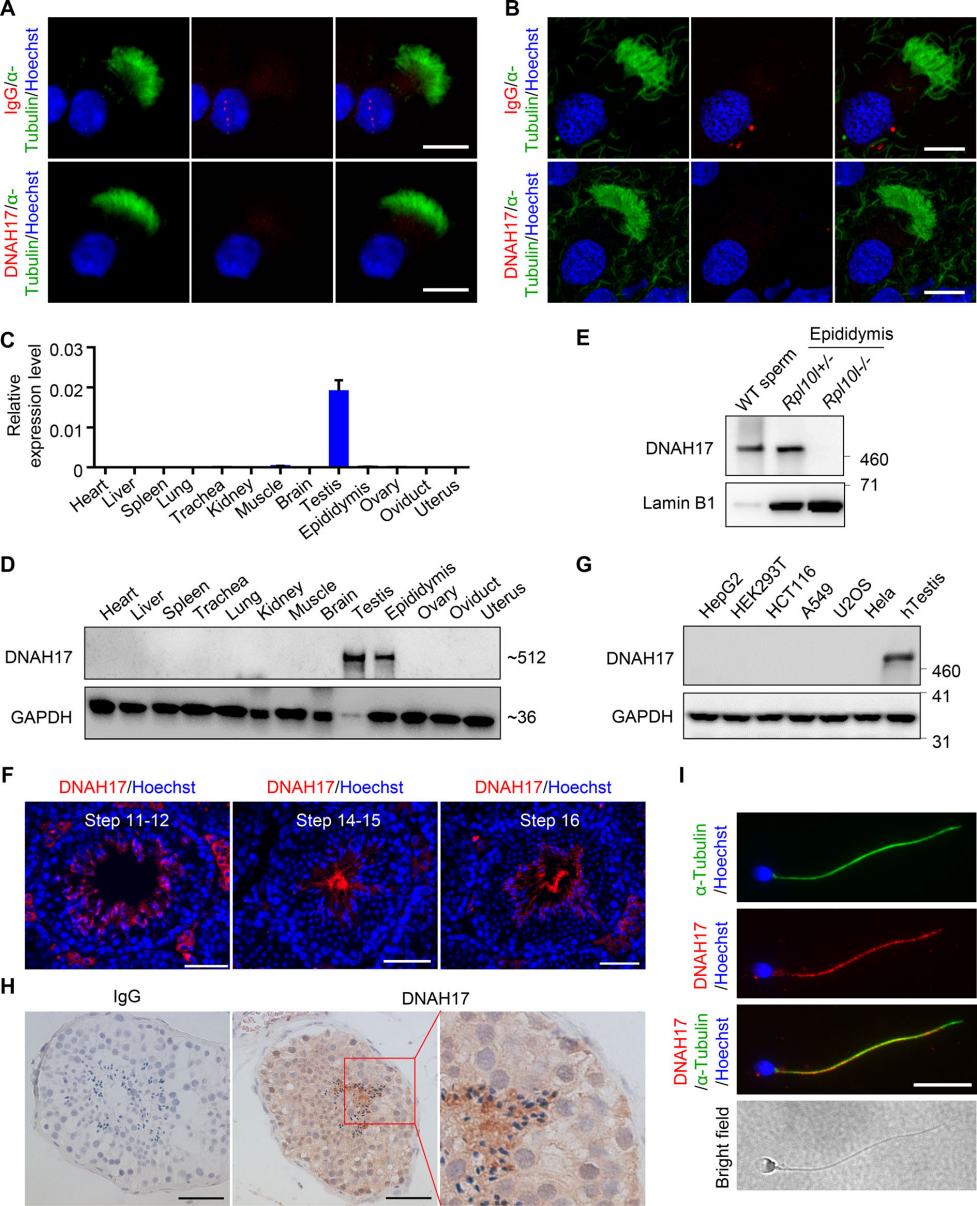
**Expression and localization of DNAH17 in humans and mice. (A and B)** Representative images of mouse (A) and human (B) respiratory cilia stained for α-tubulin (a marker for the ciliary axoneme) and rabbit IgG (negative control, upper panel) or DNAH17 (lower panel). Scale bars represent 10 µm. **(C)** Quantitative real-time PCR analysis of *Dnah17* mRNA expression in adult mouse tissues. *Actb* was used as an internal control. **(D)** Immunoblotting analysis of DNAH17 protein in different tissues from adult mice. GAPDH was used as the loading control. **(E)** Immunoblotting with sperm lysates from WT mice and epididymal lysates from *Rpl10l^+/−^* and *Rpl10l^−/−^* mice using the anti-DNAH17 antibody. Lamin B1 was used as the loading control. **(F)** Representative images of testicular tubules stained with anti-DNAH17 antibody and Hoechst showing that DNAH17 is localized in the cytoplasm and flagella of step 11–16 spermatids. Scale bars represent 50 µm. **(G)** Immunoblotting with lysates of human cell lines HepG2 (from liver), HEK293T (from embryonic kidney), HCT116 (from colon), A549 (from alveolar basal epithelia), U2OS (from bone), HeLa (from cervix), and adult human testes (hTestis) using the anti-DNAH17 antibody. GAPDH was used as the loading control. **(H)** Immunohistochemistry using the anti-DNAH17 antibody on adult human testicular sections with normal spermatogenesis. Rabbit IgG (left panel) was used as a negative control. Scale bars represent 50 µm. **(I)** Representative images of spermatozoa from fertile men (controls) stained with anti-DNAH17 antibody, anti–α-tubulin antibody, and Hoechst. Scale bar represents 10 µm. For A–I, three independent experiments were performed.

**Figure S2.**
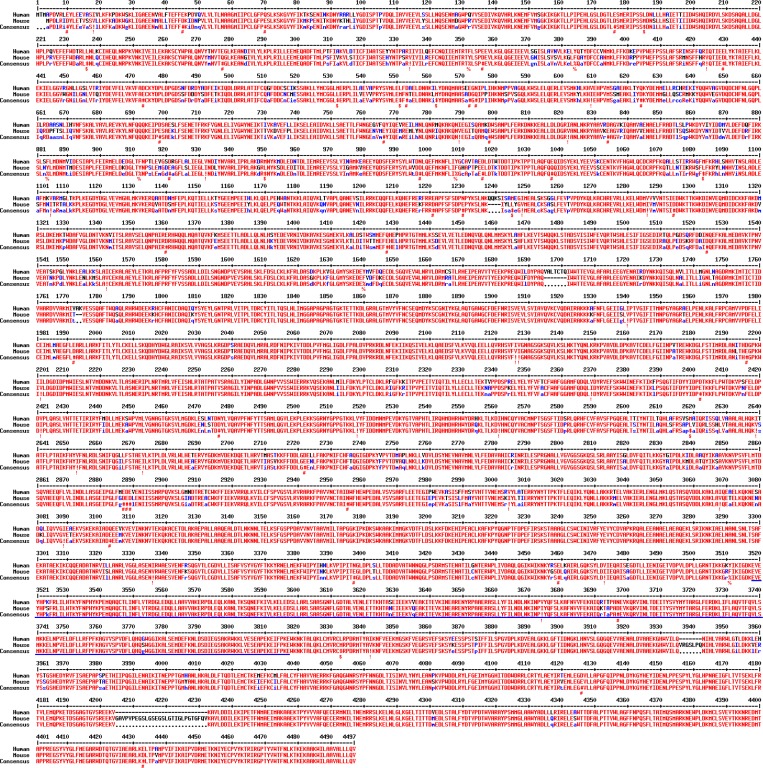
**Alignment of human and mouse DNAH17 protein sequences showing that 91% of amino acids are identical.** Residues that are identical between human and mouse DNAH17 appear in red and as uppercase letters in the consensus line. Residues highly similar between human and mouse DNAH17 are indicated by red symbols (!, any one of I and V; $, any one of L and M; %, any one of F and Y; #, any one of N, D, Q, E, B, and Z). Unconserved residues are written in blue or as asterisks in the consensus line. Blue lines highlight the epitope (amino acids 3502–3801 for mouse DNAH17, corresponding to amino acids 3518–3817 in human DNAH17) for antibody generation. The alignment was performed using the online software MultAlin (http://multalin.toulouse.inra.fr/multalin/multalin.html).

**Figure S3.**
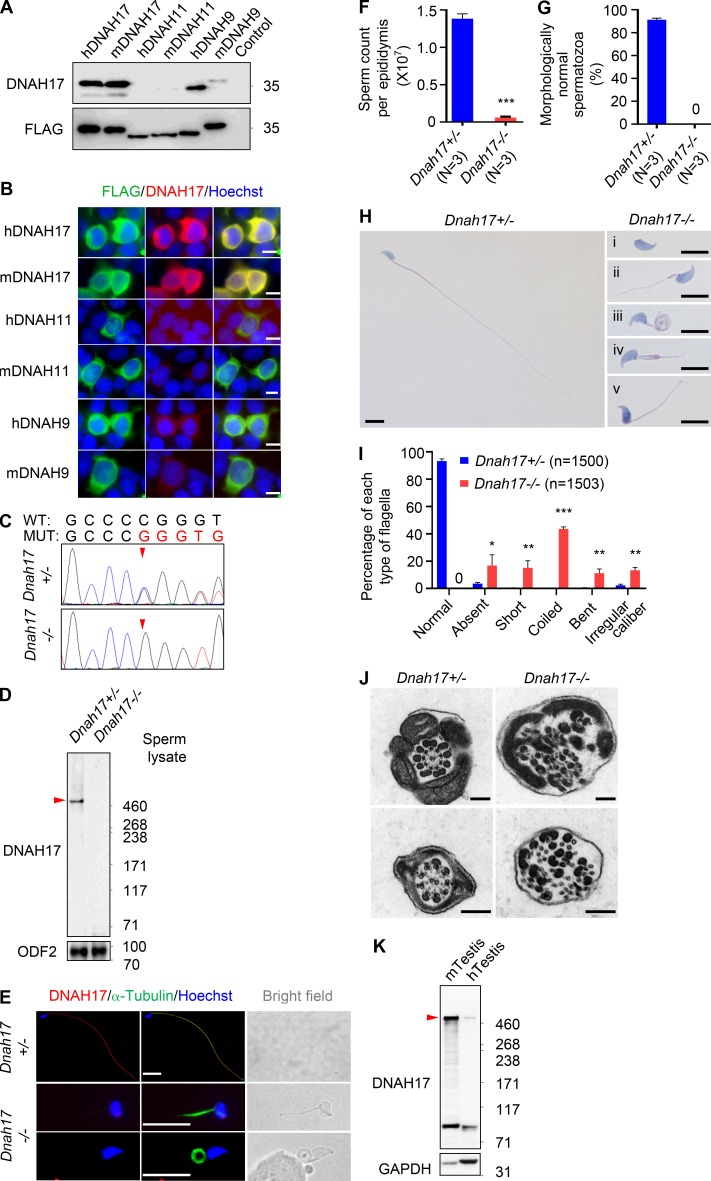
**Validation of the anti-DNAH17 antibody in transfected cells and *Dnah17* knockout mice. (A and B) **Immunoblotting (A) and IF staining (B) of HEK293T cells overexpressing the epitope-corresponding peptides from human DNAH17 (amino acids 3518–3817), mouse DNAH17 (amino acids 3502–3801), human DNAH11 (amino acids 3572–3871), mouse DNAH11 (amino acids 3544–3843), human DNAH9 (amino acids 3542–3841), or mouse DNAH9 (amino acids 3540–3839) with an N-terminal FLAG tag. For immunoblotting, untransfected cells were used as negative control. Two independent experiments were performed. Scale bars represent 10 µm. **(C)** Genomic DNA sequencing chromatograms showing the one-nucleotide-deletion mutation (g.971_971del) in *Dnah17^−/−^* mice. Arrowheads indicate the mutation site. WT, the wild-type allele. MUT, the mutant allele. **(D)** Immunoblotting using the anti-DNAH17 antibody detected a specific band at the predicted size of DNAH17 (512 kD), as indicated by the arrowhead, in spermatozoa from *Dnah17^+/−^* mice, but not in spermatozoa from *Dnah17^−/−^* mice. ODF2 (a marker of outer dense fibers) was used as the loading control. **(E)** Representative images of spermatozoa from *Dnah17^+/−^* and *Dnah17^−/−^* mice stained for DNAH17 and α-tubulin, a marker for sperm flagellum. Scale bars represent 10 µm. **(F)** Sperm count per epididymis in *Dnah17^−/−^* mice. **(G)** Quantification of the spermatozoa with normal morphology. **(H)** Representative images of spermatozoa after Papanicolaou staining showing absent (i), short (ii), coiled (iii), bent (iv), and irregular-caliber (v) flagella in *Dnah17^−/−^* mice. Scale bars represent 5 µm. **(I)** Frequencies of sperm flagella that were morphologically normal, absent, short, coiled, bent, or of irregular caliber. Each spermatozoon was classified as only one type of flagellar morphology according to its major abnormality. **(J)** Representative TEM micrographs showing cross sections of proximal (upper panel) and distal (lower panel) regions of sperm flagella from *Dnah17^+/−^* and *Dnah17^−/−^* mice. Scale bars represent 200 nm. **(K)** Immunoblotting using the anti-DNAH17 antibody detected a specific band of predicted size (510 kD for human DNAH17 and 512 kD for mouse DNAH17), as indicated by the arrowhead, in testicular lysates from adult WT mice (mTestis) or fertile men (hTestis). GAPDH was used as the loading control (GAPDH was predicted to be 35.8 kD in mouse and 36.1 kD in human). For A–K, at least two independent experiments were performed. **(F, G, and I)** Data are presented as mean ± SEM. *P < 0.05, **P < 0.01, ***P < 0.001; Student’s *t* test. N, number of mice examined; n, number of spermatozoa examined.

To determine the expression and localization of DNAH17, we generated an antibody recognizing an epitope of DNAH17 amino acids 3502–3801, which is highly conserved between mouse and human (Fig. S2). To test the specificity of this antibody, immunoblotting and immunofluorescence (IF) staining assays were performed with HEK293T cells overexpressing FLAG-tagged epitope-corresponding peptides from mouse and human DNAH17, DNAH9, and DNAH11, which are the mammalian homologues of *Chlamydomonas *reinhardtii **ODA β-HCs (Pazour et al., 2006) and have high amino acid sequence similarity. The anti-DNAH17 antibody showed high affinity to the epitope-corresponding peptides of both mouse and human DNAH17 as expected. However, it also recognized the overexpressed epitope-corresponding peptides of mouse and human DNAH9, as well as mouse DNAH11, in immunoblotting assays (Fig. S3 A), and mouse and human DNAH9 and DNAH11 in IF staining (Fig. S3 B). To determine whether this anti-DNAH17 antibody recognizes the endogenous DNAH9 and DNAH11 full-length proteins in humans and mice, we performed IF staining of respiratory cilia where DNAH9 and DNAH11 have been reported to be expressed (Dougherty et al., 2016; Fliegauf et al., 2005). This anti-DNAH17 antibody yielded weak signals that were not distinguishable from those of rabbit control IgG (Fig. 2, A and B), indicating that DNAH17 is not expressed in respiratory cilia, and that this anti-DNAH17 antibody is not likely to recognize endogenous DNAH9 and DNAH11 in humans and mice.

The specificity of the antibody was further validated in *Dnah17^−/−^* mice that were generated using the CRISPR/Cas9 technique (Fig. S3 C). Immunoblotting using the antibody against DNAH17 detected a specific band at the predicted size of DNAH17 protein in sperm lysates from *Dnah17^+/−^* mice, but not in sperm lysates of *Dnah17^−/−^* mice (Fig. S3 D). Similarly, IF staining of spermatozoa from *Dnah17^+/−^* mice showed clear and specific signals of DNAH17 colocalizing with α-tubulin, one of the major constituents of microtubules (Amos and Klug, 1974), along sperm tails except the distal tip, but the signals of anti-DNAH17 antibody were completely absent in sperm flagella from *Dnah17^−/−^* mice (Fig. S3 E). Noticeably, knockout of *Dnah17* drastically reduced sperm count and resulted in morphologically abnormal spermatozoa with a typical human MMAF phenotype (Fig. S3, F–I). The 9+2 axonemal configuration was completely disrupted in flagella of *Dnah17^−/−^* mice (Fig. S3 J).

These in vitro and in vivo studies collectively indicated that, although the anti-DNAH17 antibody could cross-react with the epitope-corresponding peptides of DNAH9 and DNAH11 overexpressed in cultured cells, it is not likely to recognize endogenous DNAH9 and DNAH11 proteins in mice and humans.

### The expression and localization of DNAH17

In mice, *Dnah17* mRNA was abundantly expressed in testes but not detected in lungs, tracheae, or oviducts (Fig. 2 C). Using the antibody against DNAH17, we found that DNAH17 protein was indeed not detected in cilia from tracheae and was detected only in testes and epididymides of WT mice (Fig. 2, A, D, and E), but not in epididymides of *Rpl10l^−/−^* mice devoid of spermatozoa (Fig. 2 E; Jiang et al., 2017), indicating that DNAH17 expression is restricted to testes and spermatozoa. IF staining further showed that DNAH17 is localized predominantly in cytoplasm and flagella of step 11–16 spermatids of adult mice (Fig. 2 F). Consistently, in humans, DNAH17 was detected only in testes, but not in various somatic cell lines (Fig. 2 G and Fig. S3 K). Immunohistochemical staining on human testicular sections with normal spermatogenesis revealed that DNAH17 could be detected in cytoplasm and flagella of elongated spermatids (Fig. 2 H). IF staining on human semen smears showed localization of DNAH17 in sperm flagella (Fig. 2 I). Together, the expression and localization patterns of DNAH17 propose its potential role in spermatozoa.

### Localization of the mutant DNAH17 in patients

**Figure 3.**
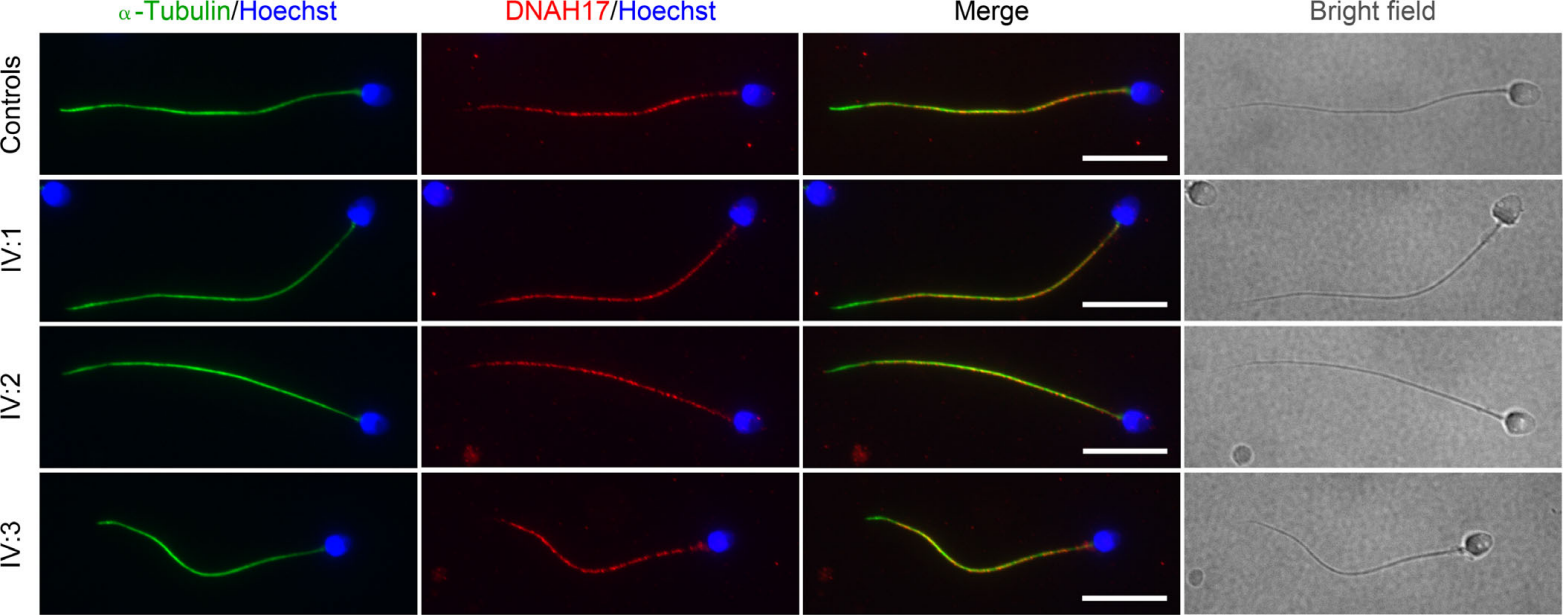
**Localization of the mutant DNAH17 is not altered in patients.** Representative images of spermatozoa from fertile controls and three patients stained with the anti-DNAH17 antibody, anti-α-tubulin antibody, and Hoechst. Two independent experiments were performed, and at least 150 sperm were examined for each time per individual. Scale bars represent 10 µm.

To explore whether the identified variant affected expression and localization of DNAH17 in patients, we performed IF staining on semen smears using the anti-DNAH17 antibody. The signals of DNAH17 and α-tubulin were detected in sperm flagella of all three patients and were not distinguishable from those in the fertile controls (Fig. 3).

### Morphological and ultrastructural analyses of sperm flagella from patients

**Figure S4.**
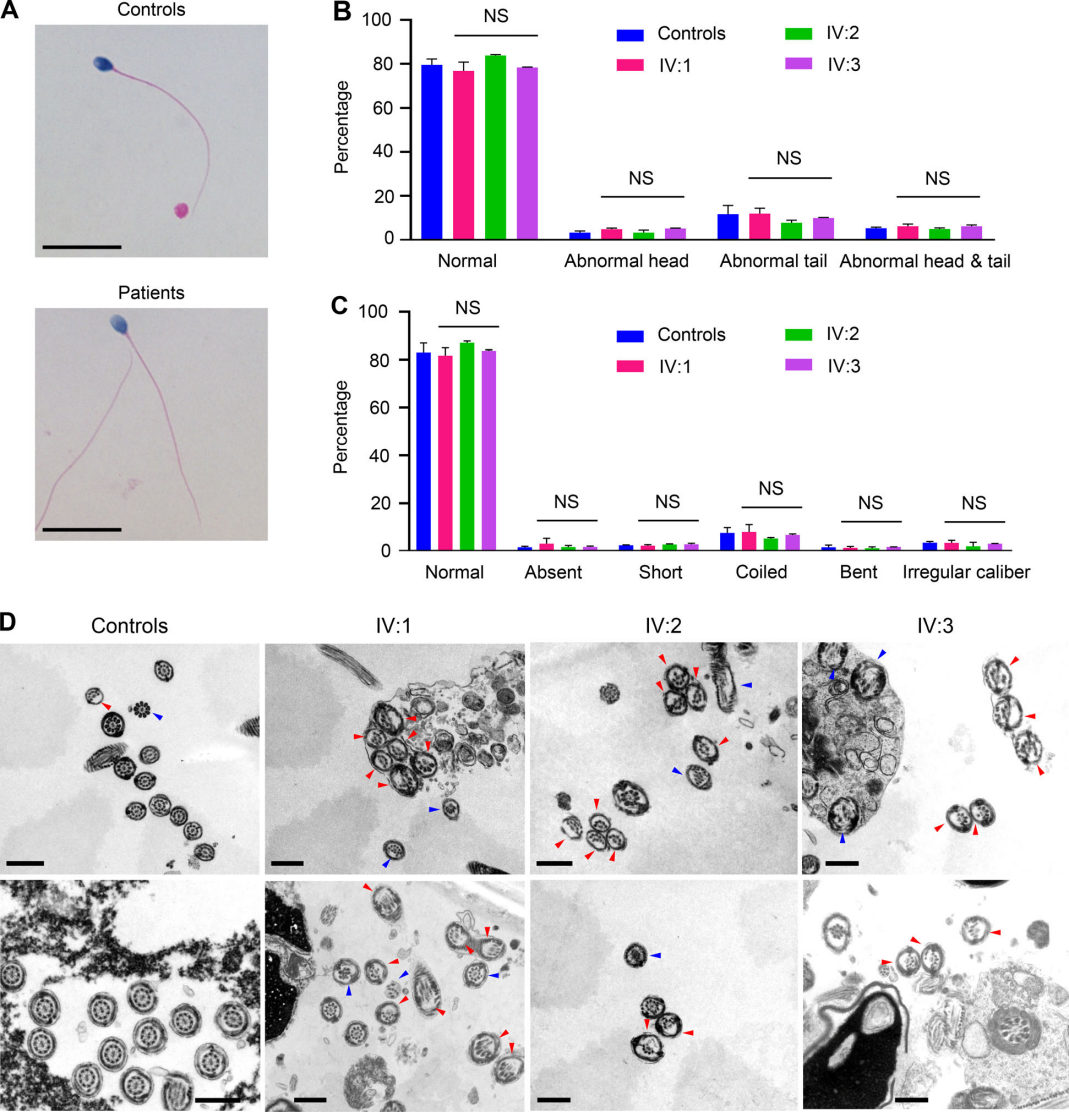
**Morphological and axoneme ultrastructural analyses of spermatozoa from patients.** **(A)** Representative images of spermatozoa from the fertile controls and patients after Papanicolaou staining. Scale bars represent 10 µm. **(B)** Quantification of spermatozoa with normal morphology, or morphological defects in sperm head, tail, or head and tail. **(C)** Frequencies of sperm flagella that are morphologically normal, absent, short, coiled, bent, or of irregular caliber. Each spermatozoon was classified as only one type of flagellar morphology according to its major abnormality. For A and B, two independent experiments were performed with at least 500 spermatozoa examined per person each time. Data are presented as mean ± SEM. Compared with the fertile control, one-way ANOVA with Dunnett‘s multiple comparison test. Two independent experiments were performed. **(D)** Representative TEM micrographs showing cross sections of sperm flagella at low magnification from fertile men (controls) and the three patients. Red arrowheads, cross sections with MTD(s) 4–7 missing; blue arrowheads, cross sections with abnormalities other than the loss of MTD(s) 4–7. Scale bars represent 500 nm.

Since mutations in *DNAH1*, another member of the DNAH family, have been reported to be associated with the MMAF phenotype (Coutton et al., 2018; Ben Khelifa et al., 2014; Sha et al., 2017; Tang et al., 2017; Wang et al., 2017), to understand whether this *DNAH17* variant could also induce an MMAF-like phenotype, we conducted further examinations of sperm morphology for all three patients. Semen samples from a fertile man with normal spermogram were used as the control. There was no significant difference in the percentage of abnormalities in sperm head, tail, or head and tail between patients and control (Fig. S4, A and B). Moreover, spermatozoa were classified into six subtypes according to flagellar morphology, including normal, absent, bent, short, coiled, and irregular-caliber flagella (Ben Khelifa et al., 2014). The flagella of >80% of spermatozoa in the patients were morphologically normal, and the frequency of each type of flagella in the patients did not significantly differ from that in the control (Fig. S4 C). Hence, all three patients did not present with the MMAF phenotype.

**Figure 4.**
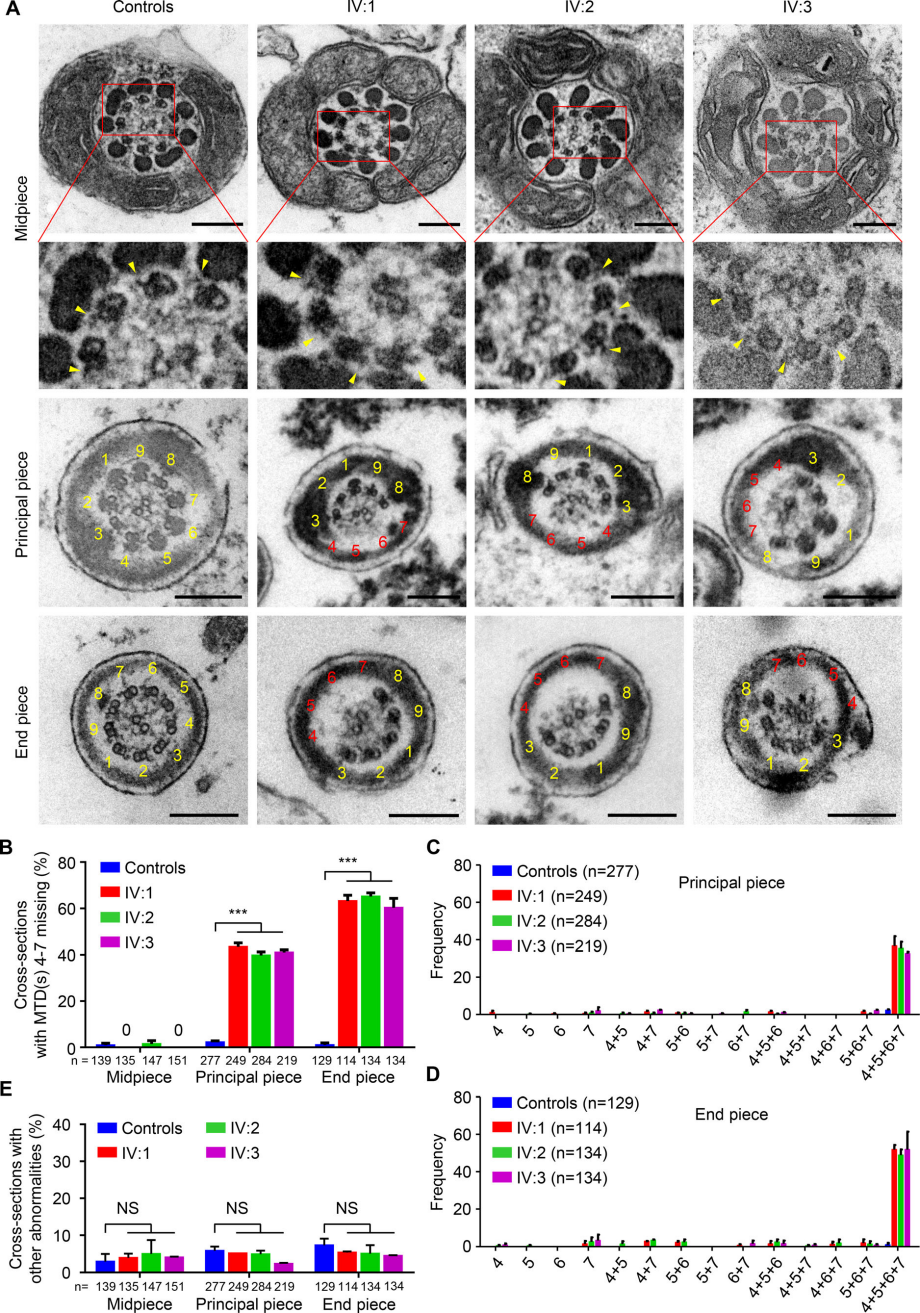
**Sperm flagella from patients show frequent absence of MTDs 4–7 at principal piece and end piece. (A)** Representative TEM micrographs showing cross sections of midpiece, principal piece, and end piece of sperm flagella from fertile men (controls) and three patients. Numbers in yellow indicate the MTDs with typical arrangement, numbers in red indicate the missing MTDs, and arrowheads highlight the ODAs. Scale bars represent 200 nm. **(B)** Quantification of flagella with loss of any combination of MTDs 4–7 at midpiece, principal piece, and end piece from controls and three patients. **(C and D)** The percentages of cross sections with MTD(s) 4, 5, 6, 7, 4+5, 4+7, 5+6, 5+7, 6+7, 4+5+6, 4+5+7, 4+6+7, 5+6+7, or 4+5+6+7 missing at principal piece (C) and end piece (D). **(E)** Quantification of cross sections with abnormalities other than the MTD(s) 4–7 missing at midpiece, principal piece, and end piece of sperm flagella from controls and three patients. Two independent experiments were performed. n, the number of axonemal cross sections analyzed. Data are presented as mean ± SEM. ***P < 0.001; one-way ANOVA test.

We next examined whether the axonemal structure of sperm tails, the horsepower apparatus that drives sperm “swimming,” was impaired by the *DNAH17* variant (Inaba, 2011; Roberts et al., 2013). Transmission EM (TEM) analyses of sperm flagella were performed. In all three patients, cross sections of midpiece displayed a typical 9+2 axonemal configuration (Fig. 4 A). However, all three patients exhibited a frequent absence of MTD(s) 4–7, accounting for ≥39.7% and ≥60.3% of all cross sections at principal piece and end piece, respectively, which were significantly higher than those in controls (2.2% at principal piece and 1.0% at end piece; Fig. 4, A and B; and Fig. S4 D). Interestingly, the associated outer dense fibers (ODFs) were also absent in the cross sections with MTD(s) 4–7 missing in our patients. Further examination of the ultrastructural anomalies in the three patients revealed that the simultaneous loss of MTDs 4–7 was most frequently observed (≥32.6% and ≥46.9% of all cross sections at principal piece and end piece, respectively; Fig. 4, A, C, and D). Besides missing MTD(s) 4–7, other abnormalities, such as disorganization of MTDs, excess microtubules, missing almost all the microtubules, etc., were also observed in patients at low frequencies, which were not significantly different from those in controls (Fig. 4 E). Intriguingly, the ODA was clearly observed attached to each of the nine MTDs at midpiece and to each of the remaining MTDs at principal piece and end piece in all three patients (Fig. 4 A). Taken together, these findings indicate that the impaired sperm motility in patients was likely due to the loss of MTD(s) 4–7 at principal piece and end piece of sperm flagella.

### Diminished sperm motility in *Dnah17^M/M^* mice

**Figure S5.**
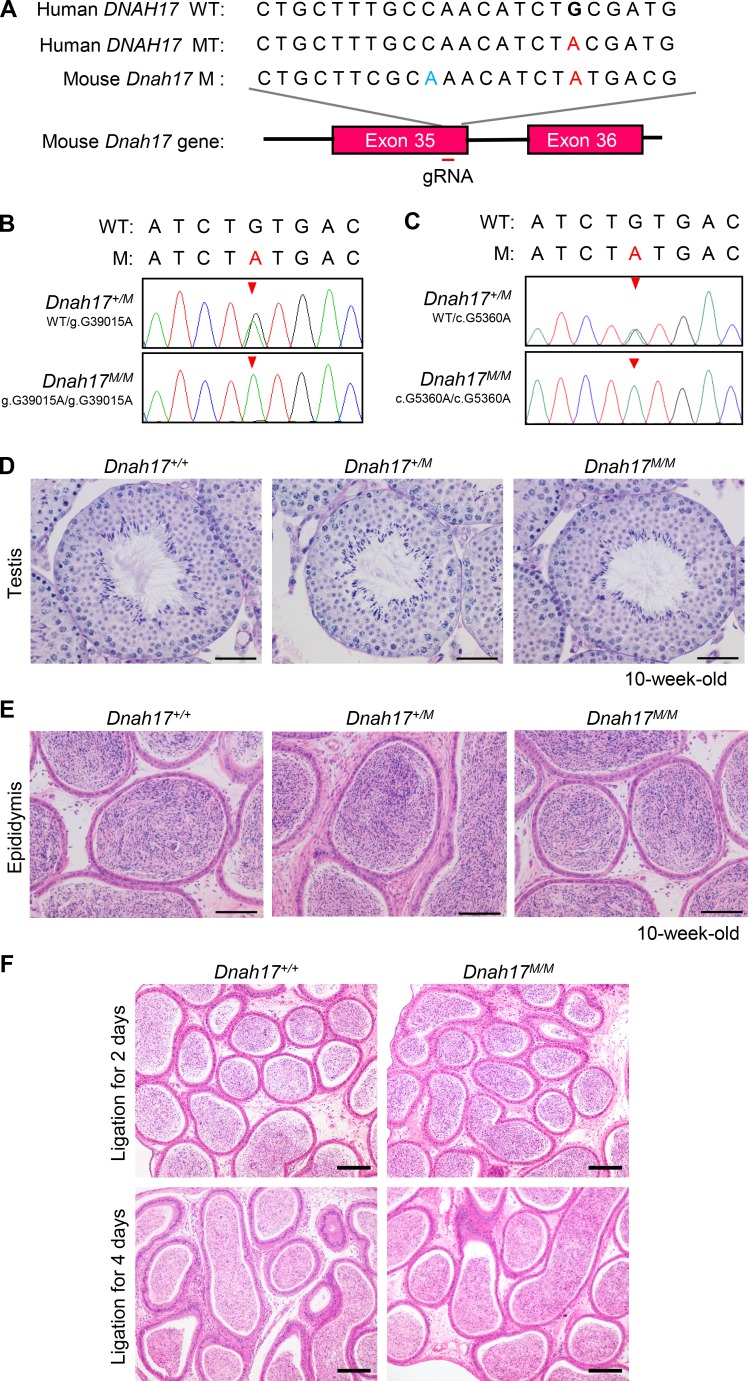
**Generation of *Dnah17^M/M^* mice modeling patients’ mutation and histological examinations of testes and epididymides from *Dnah17^+/+^*, *Dnah17^+/M^*, and *Dnah17^M/M^* mice. (A)** Schematic illustrating construction of the mouse model (*Dnah17^M/M^*). A gRNA was designed targeting exon 35 of *Dnah17.* The mutated nucleotides in mice (c.G5360A) and in patients (c.G5408A) are written in red. The nucleotide written in blue indicates a mutation not affecting the amino acid sequence in the protospacer adjacent motif, introduced by ssODNs. **(B)** Genomic DNA sequencing chromatograms showing the g.G39015A mutation heterozygous in *Dnah17^+/M^* and homozygous in *Dnah17^M/M^* mice. **(C)** cDNA sequencing chromatograms from *Dnah17^+/M^* and *Dnah17^M/M^* mice verified the c.G5360A mutation at mRNA level. **(D)** Periodic acid-Schiff staining of testicular sections showing that the spermatogenesis in *Dnah17^M/M^* and *Dnah17^+/M^* mice is comparable to that of *Dnah17^+/+^* mice. Scale bars represent 50 µm. **(E)** H&E staining of epididymal sections revealed similar sperm concentrations in *Dnah17^M/M^* and *Dnah17^+/M^* mice compared with that in *Dnah17^+/+^* mice. Scale bars represent 100 µm. **(F)** Histological examination of corpus epididymides from *Dnah17^+/+^* and *Dnah17^M/M^* mice after ligation for 2 d and 4 d. Two independent experiments were performed. Scale bars represent 100 µm. **(A–E)** Three independent experiments were performed. Arrowheads, the mutation site; WT, the wild-type allele; MT, the mutant allele in humans; M, the mutant allele in mice.

Given the 91% identity in amino acid sequence (Fig. S2) and the same expression and localization patterns of DNAH17 between human and mouse (Fig. 2), to functionally verify whether the *DNAH17* variant was indeed the pathogenic variant for the defects in patients’ sperm tails, we generated a mouse model (*Dnah17^M/M^*) that carried a homozygous *Dnah17* c.G5360A mutation (Fig. S5, A–C) equivalent to the *DNAH17* variant (c.G5408A) in patients, using CRISPR/Cas9-mediated genome editing.

*Dnah17^M/M^* male mice were subfertile, with an ∼64.8% reduction in litter size per pair compared with controls (Table 2). Further examination showed that body weight, testis weight and their ratio, testicular histology, sperm count, and sperm morphology (particularly the frequencies of morphological normal flagella and each type of abnormal flagella) in *Dnah17^M/M^* or *Dnah17^+/M^* mice were all comparable to those in *Dnah17^+/+^* mice (Table 2 and Fig. S5, D and E). The percentages of motile and progressively motile sperm showed no significant difference between *Dnah17^+/M^* mice and controls, but they were dramatically decreased in *Dnah17^M/M^* mice (Table 2). Hence, consistent with the findings in the patients (Table 1), *Dnah17^M/M^* mice displayed markedly diminished sperm motility, proving that the *DNAH17* variant is indeed pathogenic for asthenozoospermia.

**Table 2.** Characteristics of *Dnah17^+/+^*, *Dnah17^+/M^*, and *Dnah17^M/M^* male mice

Parameters	*Dnah17^+/+^*	*Dnah17^+/M^*	*Dnah17^M/M^*
Body weight (g)	27.0 ± 0.5	26.1 ± 1.0^a^	26.3 ± 0.8^a^
Testis weight (mg)	164.5 ± 3.3	167.4 ± 0.4^a^	176.2 ± 8.9^a^
Testis/body weight ratio (10^−3^)	6.1 ± 0.1	6.4 ± 0.2^a^	6.7 ± 0.2^a^
**Fertility**			
No. of fertile/total mice	3/3	3/3	5/5
Pups per fertile pair	24.7 ± 0.3	24.2 ± 0.2^a^	8.7 ± 0.5***
**Semen parameters**			
Sperm count (10^7^)	1.4 ± 0.1	1.4 ± 0.1^a^	1.3 ± 0.1^a^
Motile sperm (%)	76.7 ± 1.2	72.7 ± 0.9^a^	19.8 ± 3.4***
Progressively motile sperm (%)	32.7 ± 0.3	35.0 ± 1.2^a^	9.2 ± 2.0***
**Sperm morphology**			
Morphologically normal (%)	88.9 ± 0.6	89.3 ± 0.7^a^	90.4 ± 0.6^a^
Abnormal head (%)	1.9 ± 0.6	3.4 ± 0.4^a^	2.3 ± 1.0^a^
Abnormal tail (%)	5.9 ± 0.6	4.0 ± 0.7^a^	4.2 ± 0.7^a^
Abnormal head and tail (%)	3.3 ± 0.7	3.3 ± 0.5^a^	3.1 ± 0.2^a^
**Sperm flagella** **b**			
Morphologically normal (%)	90.9 ± 0.5	92.7 ± 0.8^a^	92.5 ± 0.6^a^
Absent (%)	2.4 ± 0.4	1.9 ± 0.3^a^	1.6 ± 0.3^a^
Short (%)	1.1 ± 0.4	1.0 ± 0^a^	1.1 ± 0.1^a^
Coiled (%)	3.0 ± 0.9	2.4 ± 0.7^a^	2.8 ± 0.6^a^
Bent (%)	1.0 ± 0.3	1.0 ± 0.3^a^	0.8 ± 0.1^a^
Irregular caliber (%)	1.6 ± 0.1	1.0 ± 0.3^a^	1.2 ± 0.3^a^

For fertility test, three *Dnah17^+/+^*, three *Dnah17^+/M^*, and five *Dnah17^M/M^* male mice (10 wk old) were each caged with two WT females (C57BL/6J; 10 wk old) for 90 d. For semen analysis, three 10-wk-old mice were examined for each genotype. Data are presented as mean ± SEM. ***P < 0.001; compared with the *Dnah17^+/+^* mice, one-way ANOVA with Dunnett‘s multiple comparison test.

a NS, not significant.

b Each spermatozoon was classified as only one type of flagellar morphology according to its major abnormality.

### Ultrastructure of sperm flagella from *Dnah17^M/M^* mice

**Figure 5.**
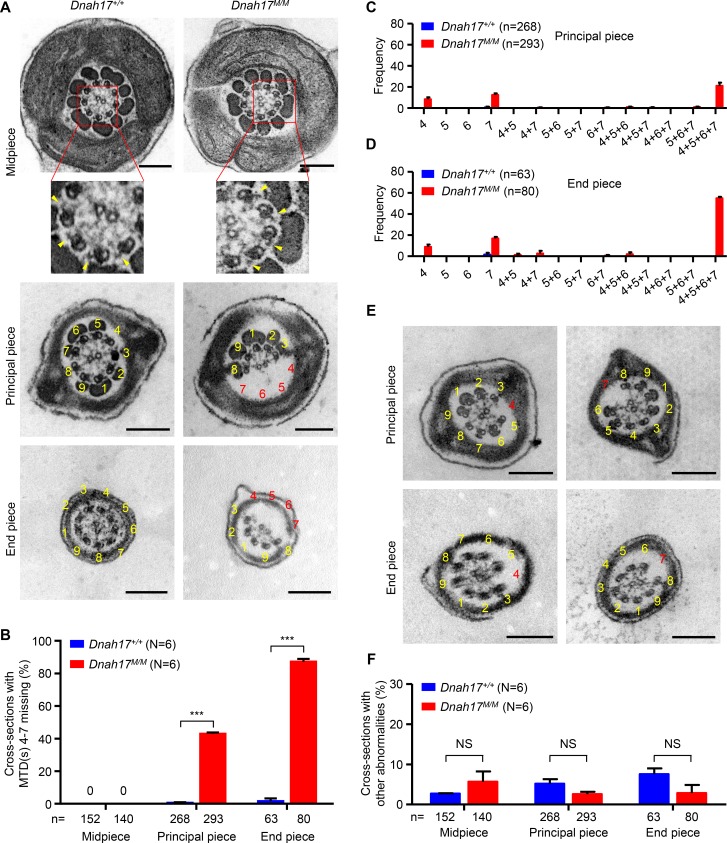
**Sperm flagella from *Dnah17^M/M^* mice show frequent absence of MTDs 4–7 at principal piece and end piece. (A)** Representative TEM micrographs showing cross sections of midpiece, principal piece, and end piece of sperm flagella from *Dnah17^+/+^* and *Dnah17^M/M^* mice. **(B)** Percentages of the flagellar cross sections with loss of any combination of MTDs 4–7 at midpiece, principal piece, and end piece. **(C and D)** Frequencies of cross sections with MTD(s) 4, 5, 6, 7, 4+5, 4+7, 5+6, 5+7, 6+7, 4+5+6, 4+5+7, 4+6+7, 5+6+7, or 4+5+6+7 missing at principal piece (C) and end piece (D) from *Dnah17^+/+^* and *Dnah17^M/M^* mice. **(E)** Representative cross sections with MTD 4 or 7 missing at principal piece and end piece of sperm flagella from *Dnah17^M/M^* mice. **(F)** Percentages of flagellar cross sections with abnormalities other than MTD(s) 4–7 missing. For A and E, numbers in yellow indicate the MTDs with typical arrangement, numbers in red indicate the missing MTDs, and arrowheads highlight the ODAs; scale bars represent 200 nm. N, the number of mice examined. n, the number of axonemal cross sections analyzed. Data are presented as mean ± SEM. ***P < 0.001; Student’s *t* test.

To understand whether the ultrastructure of sperm flagella in mice was also altered by the *DNAH17* variant, TEM analyses of flagella of spermatozoa isolated from cauda epididymides were conducted. Cross sections of midpiece from both *Dnah17^+/+^* and *Dnah17^M/M^* mice exhibited classical 9+2 axonemal configuration. Nonetheless, 42.9% and 87.2% of cross sections of principal piece and end piece, respectively, showed MTD(s) 4–7 missing with a concomitant loss of the associated ODF(s) in *Dnah17^M/M^* mice, in sharp contrast to 0.6% and 1.7%, respectively, in controls (Fig. 5, A and B). Consistent with the observations in patients, the most frequent anomaly in *Dnah17^M/M^* mice was the simultaneous absence of MTDs 4–7, accounting for 21.1% and 54.9% of all cross sections at principal piece and end piece, respectively (Fig. 5, A, C, and D). Moreover, the absence of MTD 4 or 7 was detected at lower frequencies (Fig. 5, C–E). Though some abnormalities other than the lack of MTD(s) 4–7 were also observed in flagellar cross sections of *Dnah17^M/M^* and control mice, their percentages were low and showed no significant difference (Fig. 5 F). It is also worth noting that the ODA attached to each of the remaining MTDs was visible on all the cross sections examined for *Dnah17^M/M^* mice (Fig. 5 A). Together, these TEM findings in *Dnah17^M/M^* mice recapitulated the defects of patients’ sperm tails, demonstrating that the *DNAH17* variant was indeed responsible for the absence of MTD(s) 4–7 in our asthenozoospermic patients.

### MTDs 4–7 were destabilized in cauda epididymis

**Figure 6.**
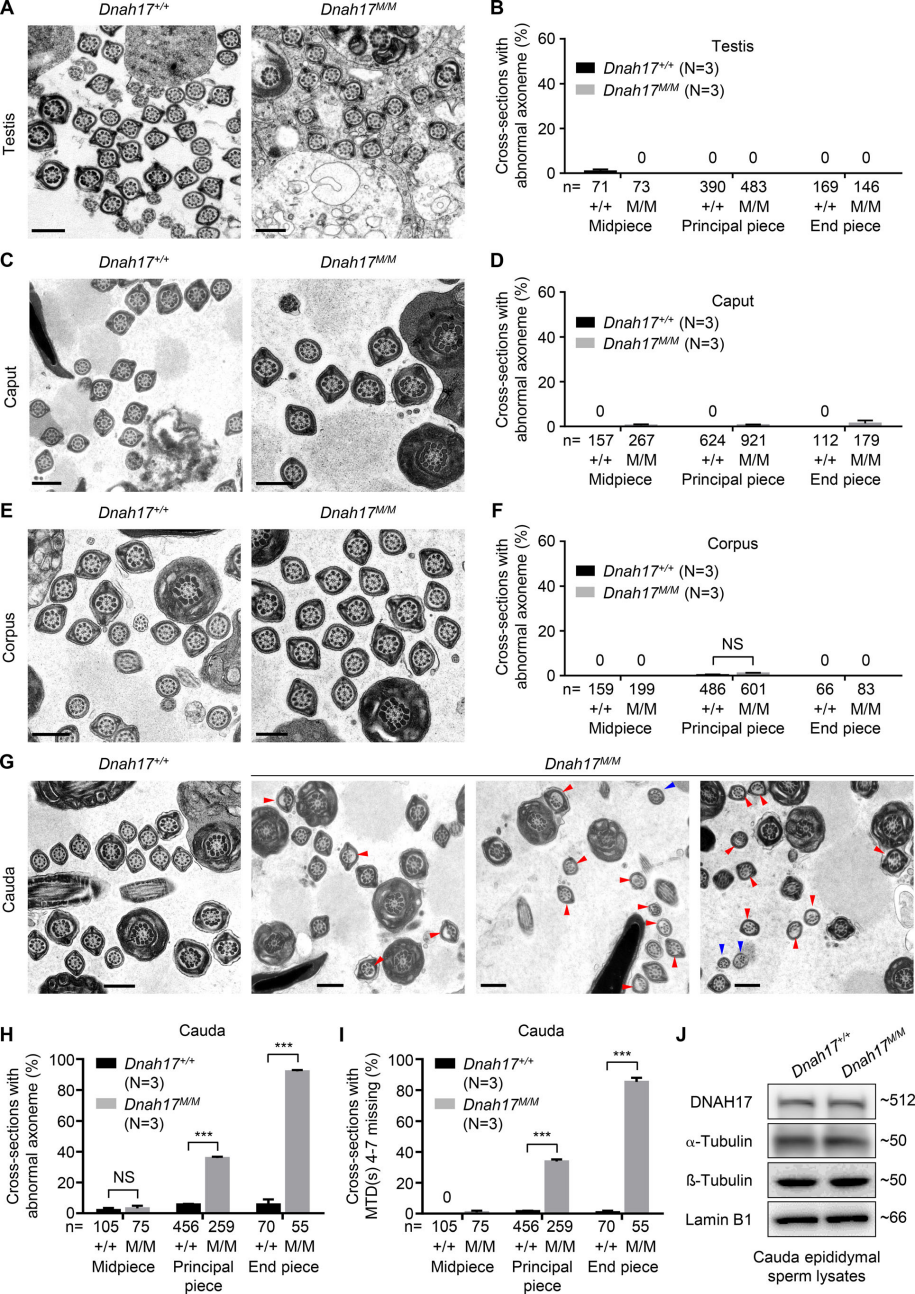
**MTDs 4–7 are destabilized in cauda epididymides of *Dnah17^M/M^* mice. (A)** Representative TEM micrographs of flagellar cross sections in testes from *Dnah17^+/+^* and *Dnah17^M/M^* mice. **(B)** The percentages of cross sections with abnormal axoneme structure (including anomalies related to MTDs 4–7 and other anomalies) in testes from *Dnah17^+/+^* and *Dnah17^M/M^* mice. **(C**–**H)** Representative TEM micrographs of flagellar cross sections and the percentages of cross sections with abnormal axoneme structure in caput (C and D), corpus (E and F), and cauda (G and H) epididymides from *Dnah17^+/+^* and *Dnah17^M/M^* mice. Red arrowheads, cross sections with loss of any combination of MTDs 4–7; blue arrowheads, cross sections with axonemal abnormalities other than the loss of MTD(s) 4–7. Scale bars represent 500 nm. **(I)** The percentages of cross sections with loss of any combination of MTDs 4–7 in cauda epididymides of *Dnah17^+/+^* and *Dnah17^M/M^* mice. **(J)** Immunoblotting with lysates of spermatozoa from cauda epididymides using anti-DNAH17, anti–α-tubulin, and anti–β-tubulin antibodies. Lamin B1 was used as the loading control. Three independent experiments were performed. N, the number of mice analyzed. n, the number of axonemal cross sections analyzed. Data are presented as mean ± SEM. ***P < 0.001; Student’s *t* test.

The frequent absence of MTD(s) 4–7 observed in spermatozoa from both patients and *Dnah17^M/M^* mice could be due to either defective flagellum biogenesis or MTD destabilization. TEM analyses of the sperm flagella in seminiferous tubules retrieved from testes were performed and revealed that abnormal axoneme structure was not observed in all cross sections examined for *Dnah17^M/M^* mice (Fig. 6, A and B), indicating that the cause of MTD(s) 4–7 absence was not a defect in axonemal assembly.

To investigate whether the lack of MTD(s) 4–7 occurred during transition of spermatozoa, we performed TEM analyses of spermatozoa within caput, corpus, and cauda epididymides and found that axonemal structure abnormality was hardly detected in all cross sections examined for *Dnah17^M/M^* caput and corpus epididymides (Fig. 6, C–F). However, flagella in *Dnah17^M/M^* cauda epididymides displayed significantly higher frequencies of abnormal axoneme structure at principal piece and end piece than in controls (Fig. 6, G and H). Interestingly, in *Dnah17^M/M^* cauda epididymides, 33.8% and 85.3% of cross sections at principal piece and end piece, respectively, presented with MTD(s) 4–7 missing, while only 1.7% and 0.9%, respectively, were detected with such abnormalities in controls (Fig. 6 I). The levels of α-tubulin, β-tubulin, and DNAH17 in lysates of sperm from cauda epididymides were further evaluated and found to be comparable between *Dnah17^M/M^* and control mice (Fig. 6 J), indicating that these proteins were not degraded and the lack of MTD(s) 4–7 could be due to destabilization. Hence, these findings revealed that the structural defects of sperm flagella observed in patients and *Dnah17^M/M^* mice could result from the destabilization of MTDs 4–7 occurring specifically in cauda epididymis.

### Destabilization of MTDs 4–7 is related to the storage of sperm in epididymides

**Figure 7.**
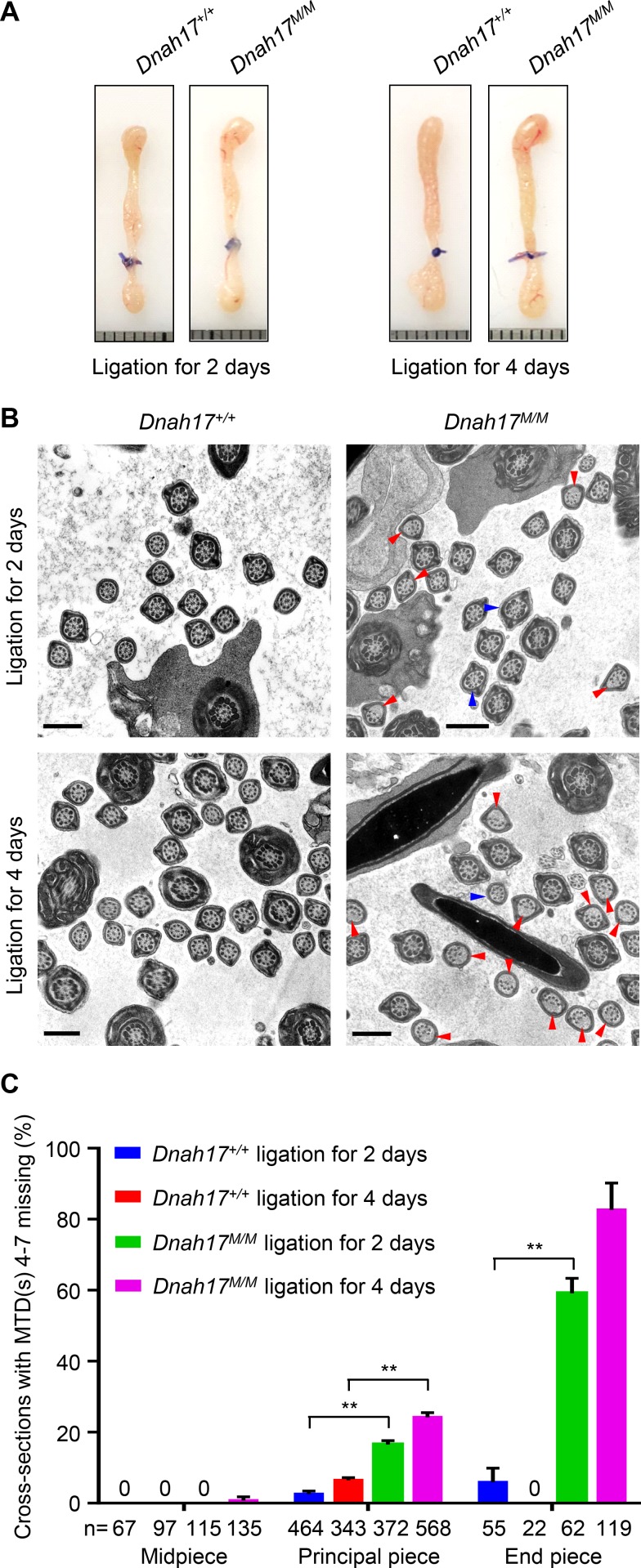
**TEM analyses of sperm flagella in corpus epididymides after epididymal duct ligation. (A)** Representative images of epididymides from *Dnah17^+/+^* and *Dnah17^M/M^* mice after ligation for 2 d and 4 d. The epididymal ducts were ligated at the end of corpus adjacent to cauda. Each grid represents 1 mm. **(B)** Representative TEM micrographs of flagellar cross sections in corpus epididymides after ligation for 2 d and 4 d. Red arrowheads indicate cross sections with loss of any combination of MTDs 4–7, and blue arrowheads indicate cross sections with axonemal abnormalities other than the loss of MTD(s) 4–7. Scale bars represent 500 nm. **(C)** The percentages of cross sections with loss of any combination of MTDs 4–7 in corpus epididymides of *Dnah17^+/+^* and *Dnah17^M/M^* mice. Two independent experiments were performed. n, the number of axonemal cross sections analyzed. Data are presented as mean ± SEM. **P < 0.01; one-way ANOVA test.

Since cauda epididymis is the place where spermatozoa are stored before ejaculation, we thus examined whether the storage time could predispose MTDs 4–7 to destabilization. We conducted epididymal duct ligation at the end of corpus adjacent to cauda for 2 d and 4 d, respectively (Fig. 7 A), simulating the different lengths of time that spermatozoa were stored in epididymides. 2 or 4 d after ligation, a large number of spermatozoa were accumulated in the corpus region in both WT and *Dnah17^M/M^* mice (Fig. S5 F). TEM analyses of flagella of sperm in the corpus were subsequently performed. Compared with WT mice, significantly increased frequencies of MTD(s) 4–7 missing at the principal piece (16.5%) and end piece (59.1%) were observed in *Dnah17^M/M^* mice 2 d after ligation (Fig. 7, B and C). 4 d after ligation, the frequencies were further increased to 24.2% at principal piece and 82.7% at end piece in *Dnah17^M/M^* mice, while the frequencies in control mice remained very low (Fig. 7, B and C). These observations indicate that MTDs 4–7 destabilization is negatively associated with the length of ligation time. Thus, combined with the findings on flagellar structure of sperm in cauda epididymides (Fig. 6, G–I), we proposed that the destabilization of MTDs 4–7 in *Dnah17^M/M^* mice is most probably due to storage of sperm in epididymides, regardless of storage in corpus or cauda.

## Discussion

Our study identifies a homozygous missense variant (c.G5408A) in *DNAH17*, a functionally uncharacterized gene, from a consanguineous Pakistani family with three offspring suffering from asthenozoospermia (no MMAF-like phenotype) and provides genetic evidence that *DNAH17* c.G5408A is pathogenic for asthenozoospermia using *Dnah17^M/M^* mice modeling the patients’ mutation. Extensive examinations of the spermatozoa from the three patients and *Dnah17^M/M^* mice collectively elucidate that the *DNAH17* variant causes frequent absence of MTD(s) 4–7 at principal piece and end piece during the sperm storage in epididymides. Thus, we demonstrate for the first time that DNAH17 is essential for sperm motility, and is the only known DNAH protein implicated in stabilizing flagellar structure, specifically MTDs 4–7.

Eukaryotic cilia and sperm flagella share a highly conserved 9+2 axonemal structure, constituted of microtubules, motor dynein arms, and their associated structures. Thus, it is not surprising that a multitude of mutations in genes encoding axonemal machinery has been identified in humans that are commonly linked to PCD and asthenozoospermia (Ji et al., 2017). All the family members in our study declared not having any ciliary-related symptoms and thus refused to participate in any further related examination. One sister (IV:5), who is homozygous for the *DNAH17* variant, had three children and no miscarriages, suggesting that *DNAH17* may be dispensable for ciliary functions. Furthermore, we found that both human and mouse DNAH17 is highly expressed in testes and spermatozoa, but was not detected in respiratory cilia, indicating that DNAH17 is required only for flagella but not cilia. Hence, we conclude that the *DNAH17* variant causes isolated asthenozoospermia without any other PCD-related symptoms.

DNAH17, along with DNAH9 and DNAH11, is a homologue of *C.* *reinhardtii* ODA β-HCs in mammals (Pazour et al., 2006). Different from the testis/spermatozoa-restricted expression pattern for DNAH17, DNAH9 and DNAH11 are localized to the distal and proximal regions of respiratory ciliary axonemes, respectively (Dougherty et al., 2016; Fliegauf et al., 2005). Besides, DNAH9 was also found localized through the sperm flagella except the distal tip (Fliegauf et al., 2005), which is similar to DNAH17 localization in sperm flagella. Biallelic mutations in *DNAH9* cause PCD with a frequent loss of ODAs at the distal, but not the proximal, regions of cilia (Fassad et al., 2018; Loges et al., 2018), and the patient carrying two homozygous missense mutations in *DNAH9* presented with oligoasthenoteratozoospermia (Fassad et al., 2018), yet the underlying ultrastructural anomalies and pathogenic mechanism remain unknown. Biallelic mutations in *DNAH11* in patients are known to cause PCD without obvious defects in the ciliary ultrastructure; however, the sperm motility, flagellar ultrastructure, and fertility status of these patients were not mentioned (Bartoloni et al., 2002; Knowles et al., 2012; Lucas et al., 2012; Pifferi et al., 2010; Schwabe et al., 2008), with the exception of one man, who had one child without the use of medical assistance (Schwabe et al., 2008). The mouse model carrying a mutation (E2271K) in *Dnah11* displayed reduced fertility and sperm motility, though the ultrastructure of sperm tails appeared normal (Lucas et al., 2012). Hence, it remains uncertain whether DNAH11 is required for male fertility. Future studies are needed to further explore the roles of DNAH11 and DNAH9 in sperm tails.

Disruptions in flagellar axoneme proteins have been reported to be associated with MMAF in humans (Martinez et al., 2018). Thus, it is not surprising that knockout of *Dnah17* induced an MMAF phenotype, which likely resulted from defective flagellar biogenesis. Noticeably, *Dnah17^M/M^* mice displayed a normal flagellar biogenesis but destabilized MTDs 4–7 during epididymal sperm storage, which is a unique phenotype that has not been reported so far in human or animal mutants for any other DNAHs (Ben Khelifa et al., 2014; Lucas et al., 2012; Neesen et al., 2001; Sha et al., 2017; Wang et al., 2017; Zuccarello et al., 2008). It is implied that the *Dnah17* missense mutation is less deleterious than *Dnah17* knockout, thus allowing the identification of the indispensable role of DNAH17 in stabilizing MTDs 4–7 during sperm storage in epididymis. We have also tried to detect whether there was a frequent loss of MTD(s) 4–7 in *Dnah17^−/−^* mice, but the flagellar axoneme structures were so disorganized that we were unable to determine the order of MTDs in *Dnah17^−/−^* mice. Taken together, the phenotypic difference between *Dnah17^−/−^* mice and our patients or *Dnah17^M/M^* mice indicates that DNAH17 is required for both flagellar biogenesis during spermiogenesis and stabilizing MTDs 4–7 during sperm storage in epididymis. It would be interesting to know whether other axoneme proteins are also implicated in stabilizing axonemal structures and their mutation frequency in asthenozoospermic patients with ultrastructure defects but normal morphology of flagella, which have not been paid enough attention in the past.

After leaving the testis, sperm entered the caput epididymis, progressing to corpus, and finally reached the cauda, where they were stored until ejaculation. Here, we found that, when sperm were kept in corpus for 2 or 4 d, MTDs 4–7 were also destabilized in *Dnah17^M/M^* mice, showing a time-dependent manner. Noticeably, at the end piece, the frequency of MTD(s) 4–7 missing in the corpus of *Dnah17^M/M^* mice with epididymal duct ligated for 4 d was similar to that in the cauda of unligated *Dnah17^M/M^* mice; however, at the principal piece, the frequency in *Dnah17^M/M^* corpus after ligation for 4 d remained significantly less than that in the cauda of unligated *Dnah17^M/M^* mice. These findings not only indicate that MTDs 4–7 at the end piece could be more susceptible to destabilization than those at principal piece, but also imply that the destabilization likely occurs from the distal region of the flagellum. There are two possible explanations for why MTD(s) 4–7 at principal piece were more prone to destabilization in the cauda of unligated mice than in the corpus after ligation for 4 d. First, the destabilization of doublets at principal piece may need a longer time than those at end piece. In unligated mice, sperm could be stored in the cauda epididymis for >4 d, as the densities of sperm in the corpus lumen 4 d after ligation (Fig. S5 F) were obviously lower than those in the cauda from unligated mice (Fig. S5 E). Second, the relatively lower pH and ionic calcium concentration and higher osmotic pressure in the lumen of cauda than in corpus (Dacheux and Dacheux, 2013; Shum et al., 2009; Turner, 2008) may also contribute to the instability of MTD(s) 4–7. Consistently, previous studies have shown that the disruption of *Pla2g3* (Sato et al., 2010), *Ttll9* (Konno et al., 2016), or *Vdac3* (Sampson et al., 2001) also induced instability of MTDs 4–7 or MTD 7 in mouse cauda epididymides. Together, these pieces of evidence led us to believe that MTDs 4–7 are different from the other MTDs, and must be armed with delicate machinery to cope with the prolonged storage and possible environmental challenges in epididymis. Therefore, future efforts should be attempted to interrogate the compositional, structural, and functional differences between MTDs 4–7 and the other MTDs. Moreover, the ultrastructural localization of DNAH17 in flagellar axoneme and interacting proteins of DNAH17 need to be elucidated in the future with antibodies suitable for use in immunoelectron microscopy and co-immunoprecipitation, to decipher the specific and interesting role of DNAH17 in stabilizing MTDs 4–7.

In conclusion, we demonstrated that a homozygous *DNAH17* missense variant specifically induces MTDs 4–7 destabilization in cauda epididymis, resulting in asthenozoospermia. It would be fascinating to determine the frequencies of *DNAH17* mutations in larger cohorts of infertile patients or patients from different ethnic backgrounds, which will have significant implications for genetic counseling, diagnosis, and ultimate treatment of asthenozoospermia, as well as uncovering novel and attractive targets for male contraceptive development.

## Materials and methods

### The participants and semen analyses

Members of a consanguineous family with three brothers suffering from infertility were recruited from a rural area of Pakistan. All three patients had routine semen analysis performed twice according to the WHO guidelines (WHO, 2010). Written informed consent forms from all participants were obtained at the beginning of the study. This study was approved by the institutional ethics committee of the University of Science and Technology of China.

### WES and linkage analysis

Genomic DNA was extracted from all the family members available, and WES of patient IV:2 and his father was performed as we previously described (Yin et al., 2019). Genome Analysis Toolkit best practice was adopted to generate variants from raw reads. Parametric linkage analysis was performed using a VCF file as previously described (Smith et al., 2011), and five regions were identified with logarithm of the odds scores >0 (Fig. S1 A). Variants within linkage regions and following Mendelian inheritance were kept for further screening.

### Filtering of candidate variants

Variants with depth >20×, genotype quality >90, and 0.5 cM interval between each other were selected as markers. A total of 3,855 genotyped singlenucleotide polymorphisms were used for linkage analysis by MERLIN with the following parameters: autosomal recessive model with disease allele frequency of 0.001 and 100% penetrance. A number of five peaks with logarithm of the odds scores >0 were identified as linkage regions. The variants located in linkage regions were annotated by ANNOVAR using the NCBI RefSeq gene annotation. We then conducted variants filtering with the following steps: (1) variants heterozygous in the father and homozygous in patient IV:2 were kept; (2) variants with minor allele frequencies >0.05, suggested by American College of Medical Genetics and Genomics for benign mutations (Richards et al., 2015), in any of the public databases, 1000 Genome project (Auton et al., 2015), ESP6500 (Fu et al., 2013), or ExAC database (Lek et al., 2016), and variants homozygous in our in-house WES variants call set generated from 578 fertile male samples (41 Pakistanis, 254 Chinese, and 283 Europeans) were excluded; (3) variants potentially affecting protein sequence and (4) in genes expressed in testis based on SpermatogenesisOnline1.0 (https://mcg.ustc.edu.cn/bsc/spermgenes/; Zhang et al., 2013) were kept; (5) variants predicted to be deleterious by less than half of the 13 software (Adzhubei et al., 2010; Choi et al., 2012; Chun and Fay, 2009; Davydov et al., 2010; Dong et al., 2015; Lindblad-Toh et al., 2011; Reva et al., 2011; Schwarz et al., 2014; Shihab et al., 2013; Shihab et al., 2015; Sim et al., 2012) covering them were excluded; (6) variants in genes for which inactivation has no effects on male fertility or spermatogenesis based on SpermatogenesisOnline1.0 (Zhang et al., 2013) were excluded; (7) the remaining variants were subsequently detected by Sanger sequencing in all the family members available (III:1, III:2, and IV:1–7). Fig. S1 B describes the flow chart of the filtering process. Sequences of primers are as follows: for *DNAH17*, forward 5′-GAC​CCT​GCC​ACT​TCC​TCT​TC-3′ and reverse 5′-AGT​CCT​TCC​AGC​CTC​CAC​AG-3′; for *GPS1*, forward 5′-TGT​CTG​GGG​ACT​GGT​GTC​CC-3′ and reverse 5′-CTG​ACC​CCC​ACG​CTT​ACC​TG-3′; for *HID1*, forward 5′-CCT​ATG​ACC​CGA​CCT​TGA​CC-3′ and reverse 5′-AGG​ACC​CAG​GGA​CGG​ATT​AC-3′; for *USP36*, forward 5′-CAG​GGA​CCT​GCG​TGC​ACT​GG-3′ and reverse 5′-GTG​CAC​ACC​CAT​GCG​GTT​CC-3′.

### RNA extraction, PCR, and quantitative real-time PCR

Tissue total RNA was extracted using Trizol reagent followed by cDNA synthesis using the PrimeScript RT reagent kit (TaKaRa, RR047A) according to the manufacturer’s protocol. PrimeSTAR HS DNA polymerase (TaKaRa, R044A) was used for PCR. The PCR reactions were performed under the following conditions: 3 min at 94°C, 40 cycles of 30 s at 94°C, 30 s at 57°C, and 30 s at 72°C. The obtained PCR products were electrophoresed on a 1.5% agarose gel, followed by Sanger sequencing. Quantitative real-time PCR was conducted with FastStart Universal SYBR Green Master (Rox; Roche, 04913850001) using a StepOne Real Time PCR System (Applied Biosystems). The PCR reactions were performed under the following conditions: 10 s at 95°C, followed by 40 cycles of 5 s at 95°C, and 30 s at 60°C. *Actb* was used as an internal control. The primers used are as follows: for Sanger sequencing of *Dnah17*-exon3-cDNA, forward 5′-CTA​CTC​GCT​GCT​AAA​CCA​GA-3′ and reverse 5′-TAG​ACG​TTT​TGT​AGG​GCT​GG-3′; for Sanger sequencing of *Dnah17*-exon35-cDNA, forward 5′-ATC​TTT​GAC​TAC​CCG​GCC​C-3′ and reverse 5′-AGG​CCC​TTG​TAG​ATG​TTT​CC-3′; for *Dnah17*-qPCR, forward 5′-ATG​ATC​ACC​GTG​GAG​AGT​TCG-3′ and reverse 5′-GAC​TGC​GTG​AGC​GTG​ATG​TA-3′; and for *Actb*-qPCR, forward 5′-ACC​AAC​TGG​GAC​GAC​ATG​GAG​AA-3′ and reverse 5′-TAC​GAC​CAG​AGG​CAT​ACA​GGG​AC-3′.

### Generation of the polyclonal anti-DNAH17 antibody

DNAH17 polyclonal antibody was generated in rabbits using amino acids 3502–3801 of mouse DNAH17 (UniProt accession no. Q69Z23) as antigens by ABclonal Biotechnology. Briefly, the 900-bp cDNA encoding the epitope was cloned into pET-28a expression vector, and the His-tagged fusion protein was expressed in *Escherichia coli*. The purified recombinant protein was used to generate polyclonal antisera in female New Zealand rabbits. Sequences of the primers used are as follows: for 8xHis-DNAH17_C300-6xHis, forward 5′-ACC​ATC​ATC​ACC​ATG​CCA​AAG​AGT​ACC​ACC​CCA​GTT​TCC​GCC​TGA-3′ and reverse 5′-TGG​TGG​TGG​TGG​TGC​TCG​AGT​TCT​TTG​GGG​AAG​ATC​TCT​TTC​TCG-3′; and for vector backbone, forward 5′-CTC​GAG​CAC​CAC​CAC​CAC​CA-3′ and reverse 5′-TTT​GGC​ATG​GTG​ATG​ATG​GTG-3′.

### Validation of the anti-DNAH17 antibody in transfected cells

HEK293T cells (ATCC, CRL-3216) were transfected with pCR3 plasmids expressing 3xFlag-tagged human DNAH17 amino acids 3518–3817, mouse DNAH17 amino acids 3502–3801, human DNAH11 (amino acids 3572–3871), mouse DNAH11 (amino acids 3544–3843), human DNAH9 (amino acids 3542–3841), or mouse DNAH9 (amino acids 3540–3839), using lipofectamine 3000 (Invitrogen, L3000015). 24 h later, immunoblotting and IF staining were performed. Sequences of primers used are: for 3xFlag-hDNAH17-C300, forward 5′-GAT​TAC​AAA​GAC​GAT​GAC​GAT​AAA​GAG​GTG​GAG​TAC​CAC​CCC​AAG​T-3′ and reverse 5′-GAT​CTA​GAG​TCG​CGG​CCG​CTC​TCC​TTG​GGG​AAG​ATC​TCC​TTC-3′; for 3xFlag-mDNAH17-C300, forward 5′-GAT​TAC​AAA​GAC​GAT​GAC​GAT​AAA​GAG​TAC​CAC​CCC​AGT​TTC​CGC​CTG​A-3′ and reverse 5′-GAT​CTA​GAG​TCG​CGG​CCG​CTT​TCT​TTG​GGG​AAG​ATC​TCT​TTC​TCG-3′; for 3xFlag-hDNAH11-C300, forward 5′-GAT​TAC​AAA​GAC​GAT​GAC​GAT​AAA​GAA​TGT​GAA​TTT​AAC​AAG​AAC​TTT​C-3′ and reverse 5′-GAT​CTA​GAG​TCG​CGG​CCG​CTT​TCT​TGA​GGT​AAT​TTT​TCT​TTT​TCT-3′; for 3xFlag-mDNAH11-C300, forward 5′-GAT​TAC​AAA​GAC​GAT​GAC​GAT​AAA​GAA​TGC​GAA​TTC​AAC​AAG​AAC​TTC​C-3′ and reverse 5′-GAT​CTA​GAG​TCG​CGG​CCG​CTT​TCT​TGC​GGT​AAC​TTT​TCT​TTT​TCA-3′; for 3xFlag-hDNAH9-C300, forward 5′-GAT​TAC​AAA​GAC​GAT​GAC​GAT​AAA​TGT​GAA​TAC​AAT​CCC​AAG​TTC​CGG​C-3′ and reverse 5′-GAT​CTA​GAG​TCG​CGG​CCG​CTC​CAC​TCC​TGT​GGG​AGC​TTC​TCT​TTC-3′; and for 3xFlag-mDNAH9-C300, forward 5′-GAT​TAC​AAA​GAC​GAT​GAC​GAT​AAA​GAG​TGT​GAA​TTC​AAT​CCC​AAG​TTC​C-3′ and reverse 5′-GAT​CTA​GAG​TCG​CGG​CCG​CTC​TCC​TGG​GGA​AAC​TTC​TCC​TTC​TCG-3′.

### Immunoblotting

The HepG2 (ATCC, HB-8065), HEK293T, HCT116 (ATCC, CCL-247), A549 (ATCC, CCL-185), U-2 OS (ATCC, HTB-96), and HeLa (ATCC, CCL-2) cells were cultured in high-glucose DMEM (HyClone, SH30022.01) supplemented with 10% FBS (GIBCO, 15140122), 100 U/ml penicillin, and 100 mg/ml streptomycin (GIBCO, 16000044). All the cultures were maintained at 5% CO_2_ at 37°C. For immunoblotting with cell lysates, cultured cells were washed with ice-cold PBS, lysed in 4X Bolt LDS Sample Buffer (Invitrogen, B0008) with NuPAGE Antioxidant (Invitrogen, NP0005), and boiled for 10 min. The cell lysates were subsequently stored at −80°C until use. Protein extracts from testes or spermatozoa from cauda epididymis were prepared using lysis buffer (50 mM Tris, pH 7.5, 150 mM NaCl, 0.5% Triton X-100, and 5 mM EDTA) containing a 1× PMSF protease inhibitor mixture (Thermo Scientific, 36978).

The proteins were then separated on a NuPAGE 3–8% Tris-Acetate Protein Gel (Invitrogen, EA03785BOX) and transferred to 0.45-µm pore-size nitrocellulose blotting membranes (GE Healthcare, 10600002) using a Mini Gel Tank (Invitrogen, A25977) electrophoresis and blotting apparatus (Tanon). Membranes were blocked with TBST buffer (50 mM Tris, pH 7.4, 150 mM NaCl, and 0.5% Tween-20) containing 5% nonfat milk for 1 h and incubated with primary antibodies diluted in TBST buffer containing 5% nonfat milk at 4°C overnight. Following incubation with secondary antibodies for 1 h, the blots were developed with chemiluminescence (ImageQuant LAS 4000, GE Healthcare). The primary antibodies that were used are mouse anti–α-tubulin (Sigma, F2168; 1:2,000), rabbit anti–Lamin B1 (Proteintech, 12987–1-AP; 1:2,000), mouse anti-GAPDH (Proteintech, 60004–1-Ig; 1:3,000), rabbit anti–β-actin (Abcam, ab8227; 1:2,000), rabbit anti–β-tubulin (Abcam, ab6046; 1:3,000), rabbit anti-ODF2 (Proteintech, 12058–1-AP; 1:1,000), and the rabbit anti-DNAH17 antibody that was custom produced by ABclonal Biotechnology. The secondary antibodies that were used are HRP-conjugated donkey anti-rabbit IgG (Biolegend, 406401; 1:10,000) and HRP-conjugated goat anti-mouse IgG (Biolegend, 405306; 1:10,000).

### IF staining

Human respiratory epithelial cells were obtained from a healthy subject by transnasal brush biopsy using disposable cytology brushes (Olympus, BC-202D-3010) and then spread onto glass slides. Mouse respiratory epithelial cells were obtained from tracheal tissues. The tracheae were cut into pieces gently, followed by centrifugation. After discarding the supernatant, the cells were resuspended in PBS containing 50% FBS and then spread onto glass slides. Human semen smears were prepared following the guideline of WHO (WHO, 2010). Mouse sperm were obtained from cauda epididymides, washed in PBS twice, and spread onto glass slides. The slides were air dried, fixed with 4% paraformaldehyde, and stored at −80°C until use.

For IF staining, slides were permeabilized with 0.1% (for sperm) or 0.2% Triton X-100 (for respiratory cells) in PBS and blocked with 3% skim milk. They were incubated with primary antibodies at 4°C overnight, followed by secondary antibodies at 37°C for 1 h, and then mounted with VECTASHIELD mounting medium (Vector Laboratories, H-1000) along with Hoechst 33342 (Invitrogen, H21492). Images of spermatozoa were captured using a Nikon ECLIPSE 80i microscope equipped with a charge-coupled device (Hamamatsu). Images of respiratory cilia were captured using the Nikon C2 Plus Confocal Laser Scanning Microscope system. The antibodies used were anti–α-tubulin (Sigma, F2168; 1:200), rabbit control IgG (Abclonal, AC005; 1:100), Alexa Fluor 488 goat anti-mouse IgG (Molecular Probes, A-21121; 1:100), and Alexa Fluor 555 donkey anti-rabbit IgG (Molecular Probes, A31572; 1:200). The customized anti-DNAH17 antibody was produced by ABclonal Biotechnology.

### Immunohistochemistry and histological analyses of testicular and/or epididymal tissues

Fresh testicular and epididymal tissues were fixed in Bouin’s solution or in 4% paraformaldehyde at 4°C overnight, followed by paraffin embedding. Paraffin-embedded tissues were sectioned (5 µm). Immunohistochemistry and IF staining of testicular sections were performed as previously described (Jiang et al., 2015; Jiang et al., 2014). The antibodies used were normal rabbit IgG (CST, 2729S; 1:100), Alexa Fluor 555 donkey anti-rabbit IgG (Molecular Probes, A31572; 1:200), and the anti-DNAH17 antibody that was custom produced by ABclonal Biotechnology. H&E and periodic acid-Schiff staining were performed for histological analyses of epididymal and testicular sections, respectively. Images were captured using a microscope (Nikon Eclipse 80i) equipped with a digital camera (Nikon DS-Ri1).

### TEM analysis

TEM was performed as previously described, with minor modifications (Yuan et al., 2015). Briefly, spermatozoa or tissues were fixed in 0.1 M phosphate buffer (PB; pH 7.4) containing 4% paraformaldehyde, 8% glutaraldehyde, and 0.2% picric acid at 4°C for at least overnight. After four washes with 0.1 M PB, samples were post-fixed with 1% OsO_4_ and dehydrated, followed by infiltration of acetone and epon resin mixture. Samples were embedded and ultrathin (70 nm) sectioned before staining with uranyl acetate and lead citrate. The ultrastructure of the samples was examined and captured by Tecnai 10 or 12 Microscope (Philips) at 100 kV or 120 kV, or by H-7650 Microscope (Hitachi) at 100 kV.

### Mouse models

*Dnah17^−/−^* mice and *Dnah17^M/M^* mice were generated by CRISPR/Cas9-mediated genome editing (Yang et al., 2013). Briefly, guide RNAs (gRNAs), targeting exon 3 (gRNA1 and gRNA2) for generating *Dnah17* knockouts or targeting exon 35 (gRNA3) for generating *Dnah17^M/M^* mice, were transcribed in vitro (Addgene, 51132). Single-strand oligodeoxynucleotides (ssODNs), with a mutation equivalent to that in patients and a synonymous mutation at the protospacer adjacent motif, was synthesized by Sangon Biotech. The gRNA1/gRNA2 or ssODNs/gRNA3 were microinjected together with Cas9 mRNAs into zygotes of B6D2F1 (C57BL/6×DBA/2J) mice (Shen et al., 2014). Genotypes of the resulting pups were determined by Sanger sequencing. The founder mice, homozygous for a missense mutation in *Dnah17* (*Dnah17^M/M^*) or heterozygous for *Dnah17* knockout (*Dnah17^+/−^*), were backcrossed onto C57BL/6 background for at least two generations, and the resulting *Dnah17^+/M^* or *Dnah17^+/−^* mice were crossed to generate *Dnah17^M/M^* or *Dnah17^−/−^* mice for our experiments. All mouse experiments were approved by the institutional animal ethics committee at the University of Science and Technology of China. The sequences of gRNAs, ssODNs, and genotyping primers are as follows: for gRNA1, 5′-TCG​AGA​CCA​TCA​TCA​TCG​AC-3′; for gRNA2, 5′-GCC​CCG​GGT​GGA​ATT​TGA​GT-3′; for gRNA3, 5′-ACA​TCT​GTG​ACG​CTC​AGA​TC-3′; for ssODNs, 5′-CTT​CAC​CTG​GCA​GTC​GCA​GCT​TCG​ACA​CCG​CTG​GGA​CGA​GGA​AAA​GAA​GCA​CTG​CTT​CGC​AAA​CAT​CTA​TGA​CGC​TCA​GAT​CAA​ATA​CTC​CTA​CGA​GTA​CCT​GGG​CAA​CAC​ACC​TCG​GCT​GGT​CAT​CAC-3′; for *Dnah17^−/−^* mouse genotyping, forward 5′-ACA​AGA​GCA​TCA​TCC​CGA​C-3′ and reverse 5′-GAT​TGC​GGT​ATC​TGG​CTC​A-3′; and for *Dnah17^M/M^* mouse genotyping, forward 5′-TGG​AAG​CCC​CTA​TCT​GTA​GC-3′ and reverse 5′-TCC​GGG​AAC​TAA​ATG​GTC​AAA-3′.

### Fertility test

A fertility test was performed by mating one 10-wk-old *Dnah17^M/M^* male with two 10-wk-old WT female mice (C57BL/6J) for 90 d. A total of five *Dnah17^M/M^*, three *Dnah17^+/M^*, and three WT male mice were tested. All the females were monitored for pregnancy. Dates of birth and numbers of pups were recorded for all the litters.

### Analyses of mouse sperm count, morphology, and motility

10-wk-old mice were sacrificed by cervical dislocation. Sperm number and motility were analyzed as previously described (Castaneda et al., 2017; Jiang et al., 2017). For sperm morphology, slides were stained by Papanicolaou staining (Solarbio, G1612) according to the manufacturer’s protocol. The percentages of morphologically normal spermatozoa were quantified according to the WHO guidelines (WHO, 2010), with at least 500 spermatozoa examined for each mouse.

### Epididymal duct ligation

Adult mice were anaesthetized by intraperitoneal administration of tribromoethanol. The epididymides of both sides were exposed through a median incision at the lower abdomen and ligated with a surgical suture at the end of the corpus adjacent to cauda. The mice were euthanized 2 or 4 d after the operation. The epididymides were removed, with one side fixed in Bouin’s for histological analyses by H&E staining and the other side fixed in 0.1 M PB containing 4% paraformaldehyde, 8% glutaraldehyde, and 0.2% picric acid for TEM analyses.

### Online supplemental material

Fig. S1 presents WES data analysis. In Fig. S2, the alignment of human and mouse DNAH17 protein sequences show that 91% of amino acids are identical. Fig. S3 presents validation of the anti-DNAH17 antibody in transfected cells and *Dnah17* knockout mice. Fig. S4 shows morphological and axoneme ultrastructural analyses of spermatozoa from patients. Fig. S5 shows generation of *Dnah17^M/M^* mice modeling patients’ mutation and histological examinations of testes and epididymides from *Dnah17^+/+^*, *Dnah17^+/M^*, and *Dnah17^M/M^* mice.
